# Role of Fe Impurity Reactions in the Electrochemical Properties of MgFeB_2_O_5_

**DOI:** 10.1021/acs.chemmater.4c02855

**Published:** 2024-12-16

**Authors:** Camilla Tacconis, Sunita Dey, Carson D. McLaughlin, Moulay Tahar Sougrati, Christopher A. O’Keefe, Iuliia Mikulska, Clare P. Grey, Siân E. Dutton

**Affiliations:** † Department of Physics, University of Cambridge, JJ Thomson Ave, Cambridge CB3 0HE, U.K.; ‡ Department of Chemistry, University of Cambridge, Lensfield Road, Cambridge CB2 1EW, U.K.; § Department of Chemistry, Advanced Centre for Energy and Sustainability (ACES), University of Aberdeen, Aberdeen AB24 3FX, U.K.; ∥ Universite de Montpellier, Institut Charles Gerhardt (UMR 5253) CC004, Place Eugene Bataillon, Montpellier, Cedex 5 FR 34095, France; ⊥ Science Division, Diamond Light Source Ltd, Harwell Science & Innovation Campus, Didcot, Oxfordshire OX11 0DE, U.K.

## Abstract

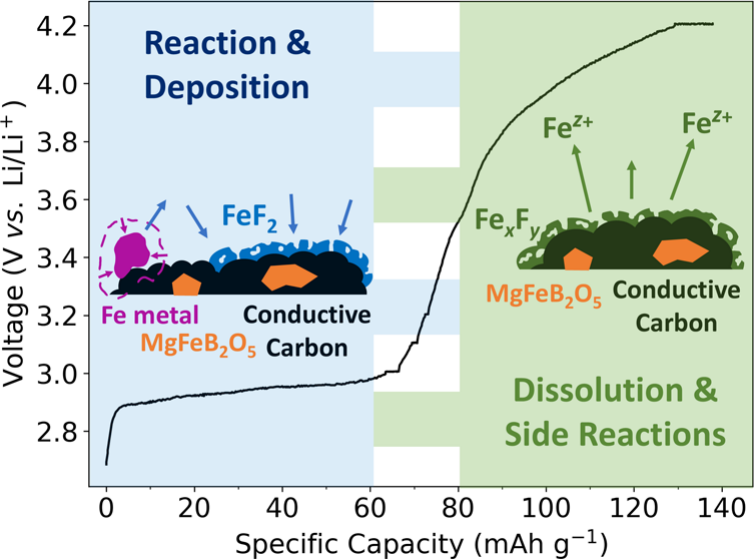

We investigate magnesium–iron pyroborate MgFeB_2_O_5_ as a potential cathode material for rechargeable magnesium-ion batteries. Synchrotron powder X-ray diffraction and Mössbauer spectroscopy confirm its successful synthesis and iron stabilization in the high-spin Fe(II) state. Initial electrochemical testing against a lithium metal anode yields a first charge capacity near the theoretical value (147.45 mAh·g^–1^), suggesting MgFeB_2_O_5_ as a promising cathode candidate. However, multimodal analyses, including scanning electron microscopy energy-dispersive X-ray (SEM-EDS) analysis, *operando* X-ray absorption near edge spectroscopy (XANES), and Mössbauer spectroscopy, reveal the absence of any Fe redox reactions. Instead, we propose that the source of the observed capacity involves the irreversible reaction of a small (4–7 wt%) Fe metal impurity. These findings highlight the need for diverse characterization techniques in evaluating the performance of new Mg cathode materials, since promising initial cycling may be caused by competing side reactions rather than Mg (de)intercalation.

## Introduction

The past decade has seen increased interest in developing renewable energies to face the issues of climate change, which has been accompanied by the necessity to develop better-performing energy storage devices.^1^ Lithium-ion batteries (LIBs) are the most commonly adopted energy storage devices at a commercial level, with applications ranging from mobile phones to battery electric vehicles.^2,3^ A growing concern in the industry regards both the safety of lithium batteries as well as the limited natural resources of Li available,^4−7^ leading researchers to develop new battery technologies to address these concerns.^8,9^ Among the next-generation rechargeable battery candidates is magnesium (Mg) ion,^10^ which presents an abundant and safer energy alternative with Mg having a high volumetric capacity (3834 mAh·cm^–3^) and low redox potential (−2.37 V vs SHE).^11^

The first cathode material to demonstrate reversible Mg storage capabilities was the Chevrel phase Mo_6_S_8_,^12−15^ reported by Aurbach et al. in 2000. To date, it has been the most successful room-temperature cathode material, demonstrating fast Mg^2+^ diffusion and good cyclability.^16^ It is used as the benchmark cathode material notwithstanding its combination of low voltages (ca. 1.1 V vs Mg^2+^/Mg)^17^ and capacities that yield low energy densities.^18^ The ongoing search for better-performing Mg cathodes has covered a wide range of materials,^19^ including binary transition metal oxides^20^ and sulfides,^21^ as well as spinels.^22,23^ While many of these studies have demonstrated reversible Mg^2+^ intercalation and good cyclability, a commercially competitive cathode material has yet to be identified.

To compete with LIB energy densities, rechargeable magnesium batteries (RMBs) need to have cathode materials that will either deliver higher specific capacities or enable high-voltage cycling of Mg^2+^ ions.^24^ This study was inspired by the commercial successes of the Li-ion analogue LiFePO_4_, where the PO_4_^3–^ polyanion enhances thermal stability, increases operating voltage, and improves reversible cycling.^25,26^ While some investigations on phosphates as Mg cathodes have been carried out,^27−29^ there is little investigation of borate candidates.^30^ MgFeB_2_O_5_, belonging to a class of polyanionic borates named pyroborates, was selected for this study due to the presence of lighter polyanion groups formed by B_2_O_5^4-^_. It promises higher voltage ranges than currently adopted Mg cathode materials thanks to the polyanion inductive effect.^31,32^

Bo et al. were the first to study Mg–Fe pyroborates as cathodes for RMBs, by analyzing the potential Mg^2+^ ionic diffusion pathways within the Mg_*x*_Fe_2–*x*_B_2_O_5_ (*x* = 2/3, 4/3) pyroborate framework.^33^ Their study observed signs of Mg^2+^ mobility at high temperatures (≈ 250 °C) through thermogravimetric analysis and identified an interstitial diffusion pathway that was reportedly insensitive to Mg/Fe site disorder. The mixed metal phase polyanion Mg_*x*_Mn_2–*x*_B_2_O_5_ (where *x* = 2/3, 1, or 4/3) was experimentally investigated by Glass et al. for Mg removal and subsequent Li cycling in a LIB. The study reported Mg^2+^ ion deintercalation in the isostructural polyanion framework at room temperature.^34^

Our work aims to resolve the debate on Mg borate polyanions as viable cathode materials by experimentally verifying Mg^2+^ mobility in MgFeB_2_O_5_ through a wide range of characterization techniques. We study the first charge behavior of the cathode material in depth, using *operando* X-ray absorption near edge spectroscopy (XANES), Mössbauer spectroscopy, and scanning electron microscopy energy-dispersive X-ray (SEM-EDS) analysis, to look for evidence of Mg mobility and Fe redox reactions. No evidence of such behavior is identified, and the promising capacity attained at the first charge is attributed to a side reaction involving a small Fe metal impurity, an unintended byproduct of our synthesis route.

## Results and Discussion

### Synthesis and Structure

**Figure 1:**
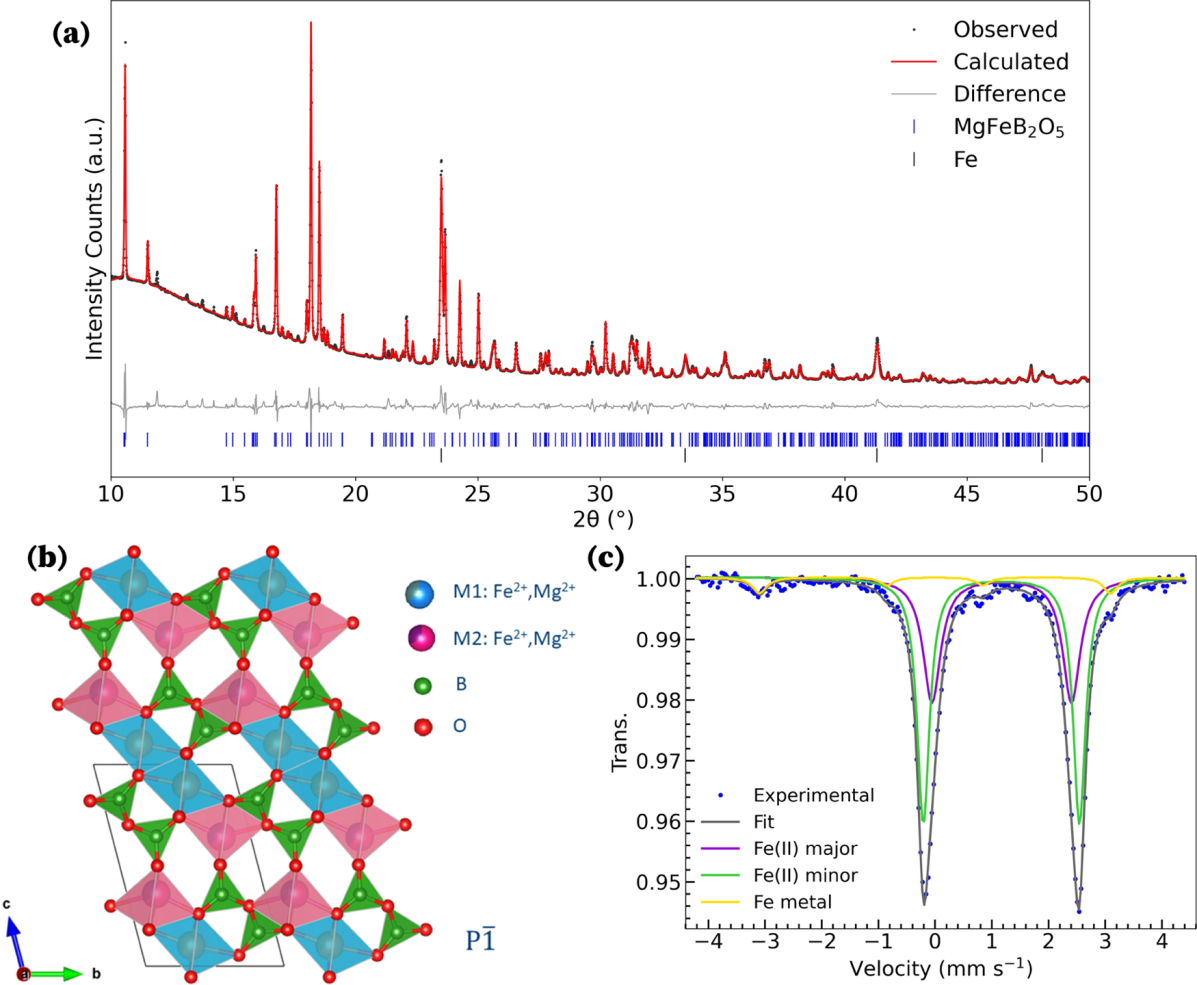
(a) Rietveld refinement of SXRD for pristine MgFeB_2_O_5_. (b) Crystal structure of MgFeB_2_O_5_ viewed along the [100]. (c) Mössbauer spectrum of pristine MgFeB_2_O_5_, with the Fe(II) major signal contributing to (58 ± 11)% of the Fe(II) species in the sample and Fe(II) minor contributing (42 ± 11)%.

The successful formation of MgFeB_2_O_5_ was verified via Synchrotron powder X-ray diffraction (SXRD). Rietveld analysis, Figure 1a indicates the presence of an ≈7 wt% Fe metal impurity. A few smaller impurities were also visible in the SXRD, but despite our best efforts, these could not be identified and are likely impurities coming from surface oxidation or contamination during synthesis.

MgFeB_2_O_5_ was shown to adopt a triclinic *P*1̅ crystal structure, as previously reported for Mg_*x*_Fe_2–x_B_2_O_5_.^33^ This structure is formed from ribbons of edge-sharing *M*O_6_ (M = Mg, Fe) octahedra interconnected via B_2_O_5_^4–^ units comprised of two corner sharing BO_3_ triangles. The *M*O_6_ octahedra form *M*_4_O_18_ tetramers that extend indefinitely along the *a*-axis. The ribbons feature a 1:1 ratio of two distinct metal sites, *M*1 (blue) and *M*2 (pink), in Figure 1b, with the external *M*2 sites characterized by greater distortion. Both sites are occupied by a disordered arrangement of Mg and Fe ions.

Mössbauer spectra were collected on the pristine pyroborate, Figure 1c. The spectrum was best fit by two Fe(II) doublets, both with an isomer shift of δ = 1.17 mm·s^–1^ and slightly different quadrupole splittings; Δ*E*_Q–major_ = 2.75 mm·s^–1^ and Δ*E*_Q–minor_ = 2.46 mm·s^–1^. The quadrupole splitting of the Mössbauer spectra is particularly sensitive to octahedral site symmetry and distortion,^35^ leading us to conclude that the two Fe(II) signals correspond to the Fe occupying the distinct *M*1 and *M*2 sites within the polyanion cathode structure. Due to the complex dependency of the quadrupole splitting of Fe(II) in an octahedral site,^36,37^ we are not able to conclude a direct correspondence between the *M*1 and *M*2 sites and the two Fe(II) Mössbauer signals. Analysis of the Mössbauer spectra also confirmed the presence of metallic Fe(0). Fe(0) is identified at a concentration corresponding to 11 at% (4 wt%) of our sample. The difference in wt% of Fe metal impurity obtained in the Mössbauer and the SXRD analyses is attributed to different synthesis iterations of the compound. We find that the amount of Fe metal present varies between 4 and 7 wt%.

### Electrochemistry

Due to the lack of suitable RMB high-voltage stable electrolytes^38,39^ and their compatibility with anode materials,^40,41^ we tested our cathode materials by cycling vs Li using LP30 electrolyte. The choice of a well-established and stable electrolyte-anode configuration was adopted to produce repeatable results that would allow for reliable analysis of MgFeB_2_O_5_ during the first charge process to investigate demagnesiation.

**Figure 2:**
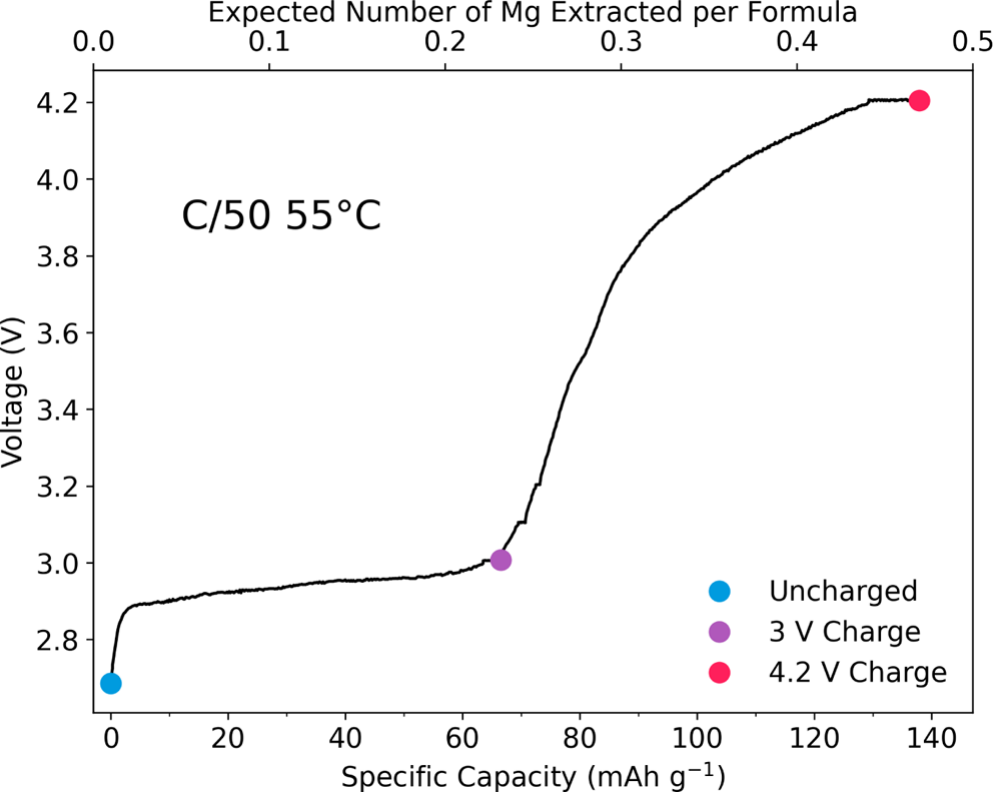
Electrochemistry of MgFeB_2_O_5_ charged to 4.2 V at a rate of C/50 at 55 °C and held at 4.2 V until the current decayed to C/200. The cathode material was tested against a Li metal anode. The number of moles of Mg extracted was calculated based on a theoretical capacity of 147.45 mAh·g^–1^. The colored dots indicate the key points in the charge profile at which postcycling analysis was performed.

The electrochemical profile is presented in Figure 2, and it shows the first charge of MgFeB_2_O_5_ against a Li metal anode. Considering the inherently slow kinetics of magnesium ions, the experiments were conducted at a low rate of C/50 and at an elevated temperature of 55 °C to optimize conditions for demagnesiation. A theoretical capacity of 147.45 mAh·g^–1^ was expected, deriving from a redox transition of Fe(II) to Fe(III) and corresponding to the extraction of 0.5 Mg^2+^ ions from the cathode structure. We observe a first charge capacity of 137.9 mAh·g^–1^, closely aligned with the expected theoretical capacity. At 2.9–3 V, a plateau is observed with a capacity of ≈60 mAh·g^–1^, which could be attributed to a two-phase deintercalation process of Mg^2+^ from the host polyanion framework. A further ≈80 mAh·g^–1^ of capacity is observed in a sloping profile between 3 and 4.2 V. The high capacity and operating voltage (4.2 V vs Li metal, ≈ 3.5 V vs Mg metal) compared to other RMB candidates, in principle, makes MgFeB_2_O_5_ a promising alternative.

Subsequent phases of the study were directed toward determining whether this observed capacity could be attributed to the demagnesiation of the cathode material. To achieve this goal, a suite of postcycling analysis techniques were applied to cathode samples at three key points in the first charge, seen in Figure 2: pristine cathode samples, samples charged up to the end of the 3 V plateau, and 4.2 V charged samples with a constant current constant voltage (CCCV) hold.

### Lack of Evidence for Fe Redox

**Figure 3:**
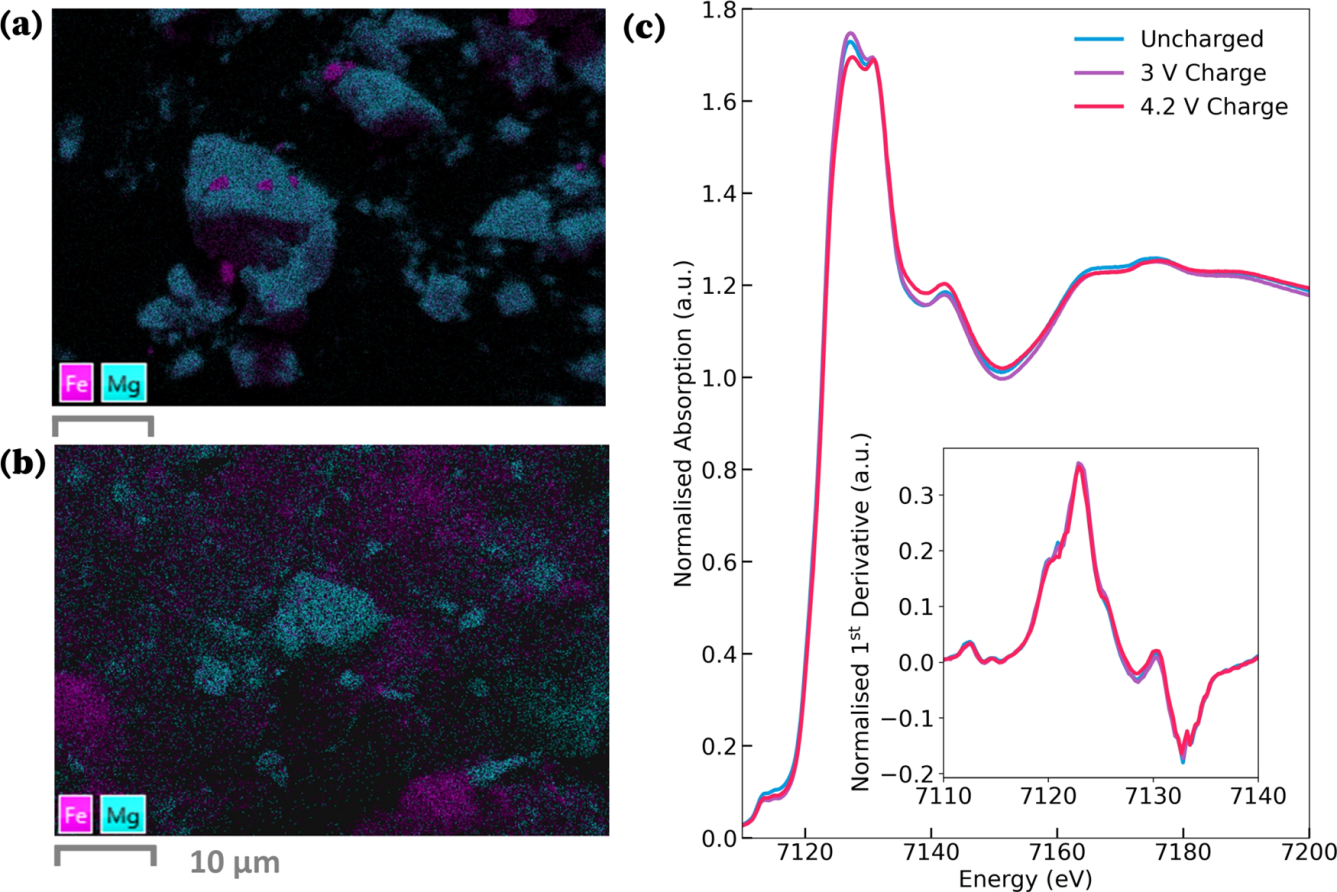
SEM-EDS maps of (a) MgFeB_2_O_5_ pristine cathode material and (b) the material charged to 4.2 V. (c) Normalized Fe K-edge *operando* XANES spectra collected at various states of charge; uncharged (blue), post 3 V plateau (purple), end of charge at 4.2 V (pink). (Inset) derivative of corresponding XANES spectra.

*Ex situ* scanning electron microscopy (SEM) was conducted on the cathode samples at various states of charge. Energy-dispersive X-ray (EDS) analysis showed no changes in the Mg content of cathode material (see Section S2 in the Supporting Information). Although this analysis did not provide evidence of demagnesiation, it facilitated the examination of the cathode’s microstructural features. Notably, we were able to determine the morphology of the iron metal impurity. The small particles are discernible in Figure 3a in pink; these are distinct and separate from the primary cathode phase. SEM maps of the cathode material after the first charge revealed the absence of these Fe particles, as shown in Figure 3b.

To examine changes in the bulk cathode, we use *operando* X-ray absorption near edge spectroscopy (XANES), which, unlike SEM, is not limited to probing surface reactions. Fe K-edge XANES spectra were collected at three points during cycling, as shown in Figure 3c. The spectra exhibit subtle variations; however, there is no detectable shift in the peak position, as evidenced in the derivative plot (inset of Figure 3c). A change in peak position would be indicative of changes in the Fe oxidation state.^42^ This lack of substantial change in Fe oxidation during the initial charging phase strongly suggests the lack of Fe redox-mediated demagnesiation from the bulk of our cathode material.

### Evidence of Fe Metal Impurity Consumption

**Figure 4:**
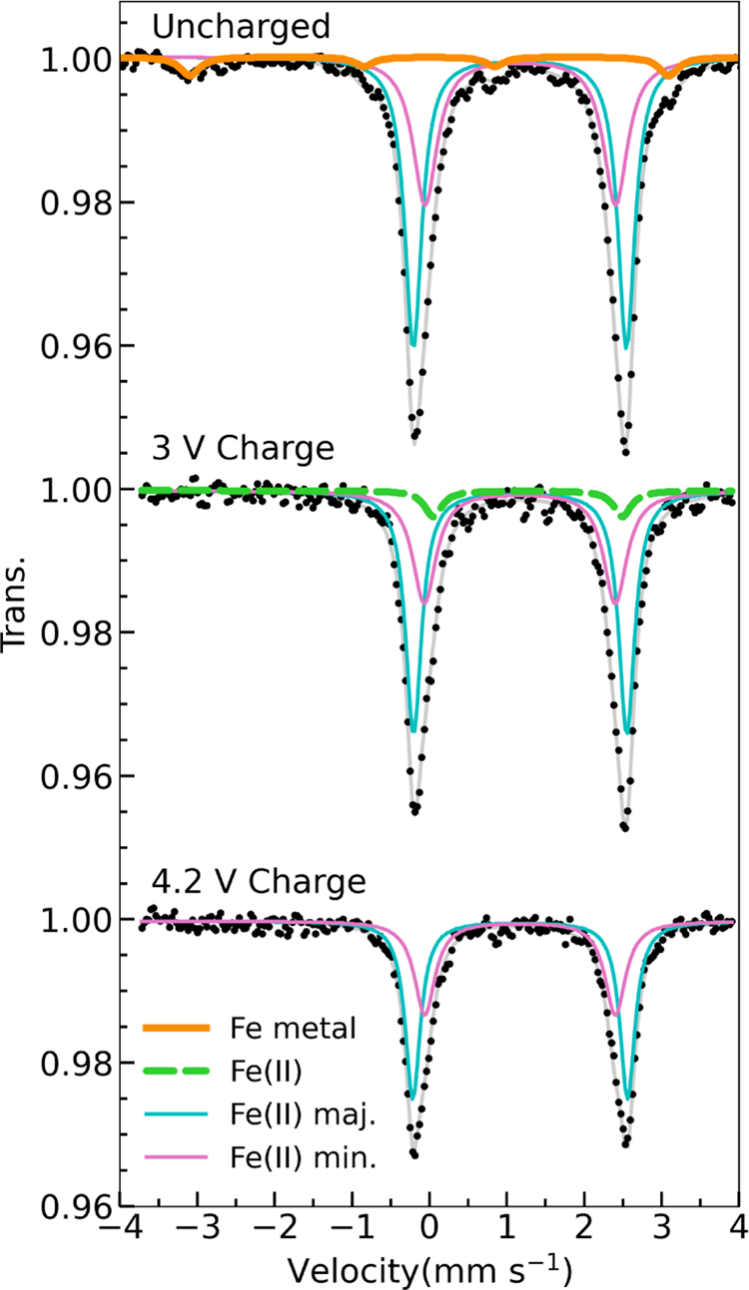
*Ex situ*^57^Fe Mössbauer spectra of MgFeB_2_O_5_ cathode samples during charge and the associated experimental fit in gray. The individual components that give rise to each fit are the main cathode phase (i) Fe(II) major (blue) and (ii) Fe(II) minor (pink), (iii) the Fe metal (orange), and (iv) the additional Fe(II) component (green). A full table with the fitted parameters can be found in Section S4 of the SI, where Figure S7 highlights the need for the third Fe(II) component in the 3 V charge fit.

Further evidence regarding the oxidation state trends of Fe in MgFeB_2_O_5_ was acquired through *ex situ*^57^Fe Mössbauer spectroscopy. The Mössbauer spectra of the charged cathode samples were experimentally fit, and the results are presented in Figure 4. In agreement with our XANES data, we find the persistence of the high-spin Fe(II) signal at all points of charge. We observe no notable changes in the isomer shift and quadrupole splitting of the Fe in the bulk cathode phase, as reported in Table S4 and seen in Figure 4 in blue and pink. A feature of interest is the presence of a third Fe(II) signal in the 3 V charged sample (in green in Figure 4), with an isomer shift of δ = 1.27(2) mm·s^–1^ and quadrupole splitting Δ*E*_Q_ = 2.44(4) mm·s^–1^. The need for this additional fitting component found only in the 3 V charged sample is discussed in Section S4 of the SI, and its interpretation is addressed later in the text. Notably, the charged samples lack the four-peak signal associated with Fe metal (in orange), indicating its consumption during the initial charging phase through an irreversible transformation (Section S7 in the SI).

**Figure 5:**
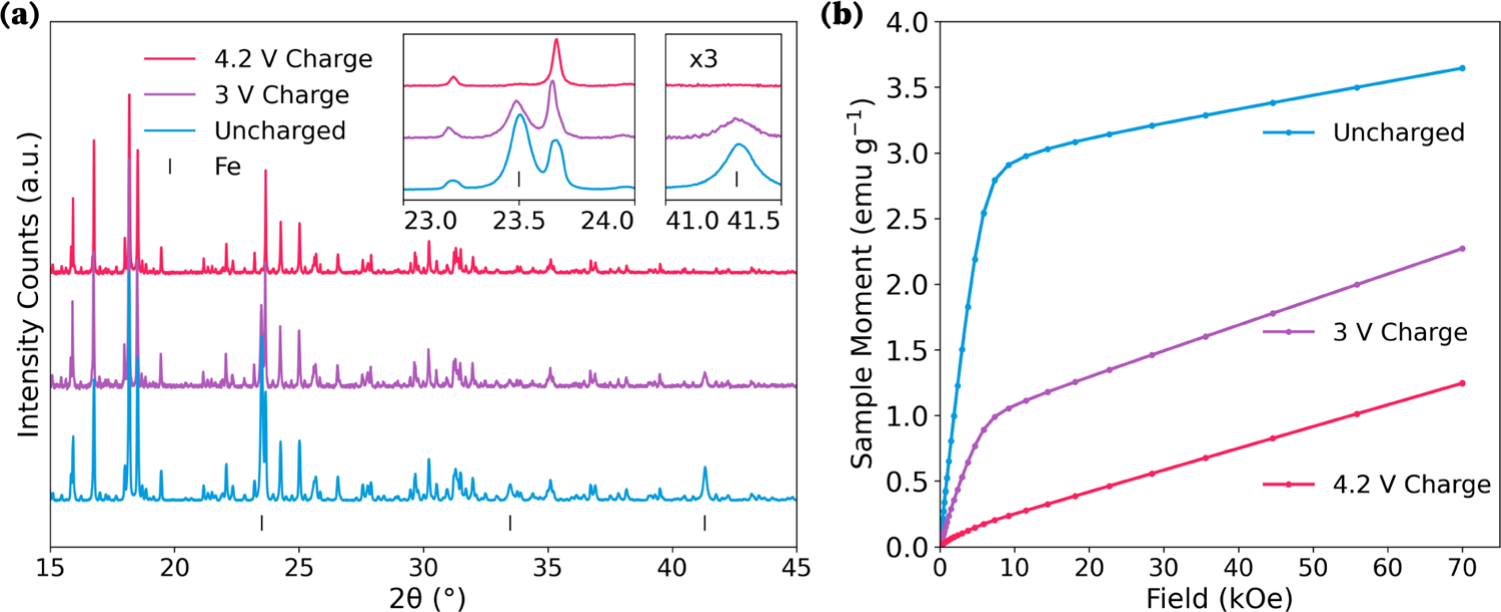
(a) Normalized Synchrotron XRD patterns for uncharged (blue), end of 3 V plateau (purple), and charged to 4.2 V (pink) cathode states. The main Fe metal peaks are indicated by the black ticks and are absent from the fully charged pattern. (Insets) Enlargement of key Fe metal peak signals. (b) M vs H curves measured at 200 K, depicting the presence of the ferromagnetic signal for the uncharged (blue) and end of 3 V plateau (purple) cathode samples and its absence in the sample charged to 4.2 V (pink).

*Ex situ* Synchrotron powder X-ray diffraction (SXRD) conducted on cathode samples extracted at the three pivotal stages of the initial charge cycle, Figure 5a, reveals no substantial modifications in the lattice parameters of the crystal structure during the first charge (Table S1 in the SI). This observed constancy does not definitively rule out demagnesiation, since an advantage of polyanion-based cathode materials is their minimal change in crystal structure during cycling.^43−45^ Notwithstanding, a key observation from the SXRD analysis is the disappearance of peaks associated with Fe metal impurity during the first charge, corroborating the findings from the Mössbauer spectra as well as the SEM-EDS.

To further explore the hypothesis of an Fe metal reaction, isothermal magnetization measurements were performed on both pristine and cycled cathode samples. The pristine cathode material exhibited a distinct ferromagnetic signal, indicative of Fe metal presence, as evidenced by the step-like feature at lower magnetic intensities, Figure 5b, which comes from ferromagnetic saturation of Fe.^46^ The magnitude of this step, which is proportional to the concentration of ferromagnetic impurities within the paramagnetic cathode material, was observed to diminish with the advancing state of charge. This trend suggests an irreversible reaction of the Fe metal impurity, leading to the formation of a nonferromagnetic product.

### Proposed Reaction Pathway

The data presented in this study show that the Fe metal impurity is undergoing an irreversible reaction throughout the first charge. Prior investigations into the behavior of iron metal current collectors in battery electrolytes have revealed that Fe produces significant corrosion currents at 3 V,^47^ consistent with a redox reaction voltage of Fe(0) to Fe(II). Here, we suggest that the capacity observed at the 3 V plateau during the initial charging cycle can be attributed to an electrochemical process involving the oxidation of the Fe metal impurity in our sample.

We can quantify the capacity contribution expected from a conversion of Fe(0) to Fe(II), taking into account the weight percentage of iron metal impurity in our samples, which ranges from 4 to 7 wt%. The calculated capacity ranges between ≈40 and 70 mAh·g^–1^, in agreement with the capacity contribution observed in the 3 V charge plateau (see Section S6 in the SI for a detailed discussion).

**Figure 6:**
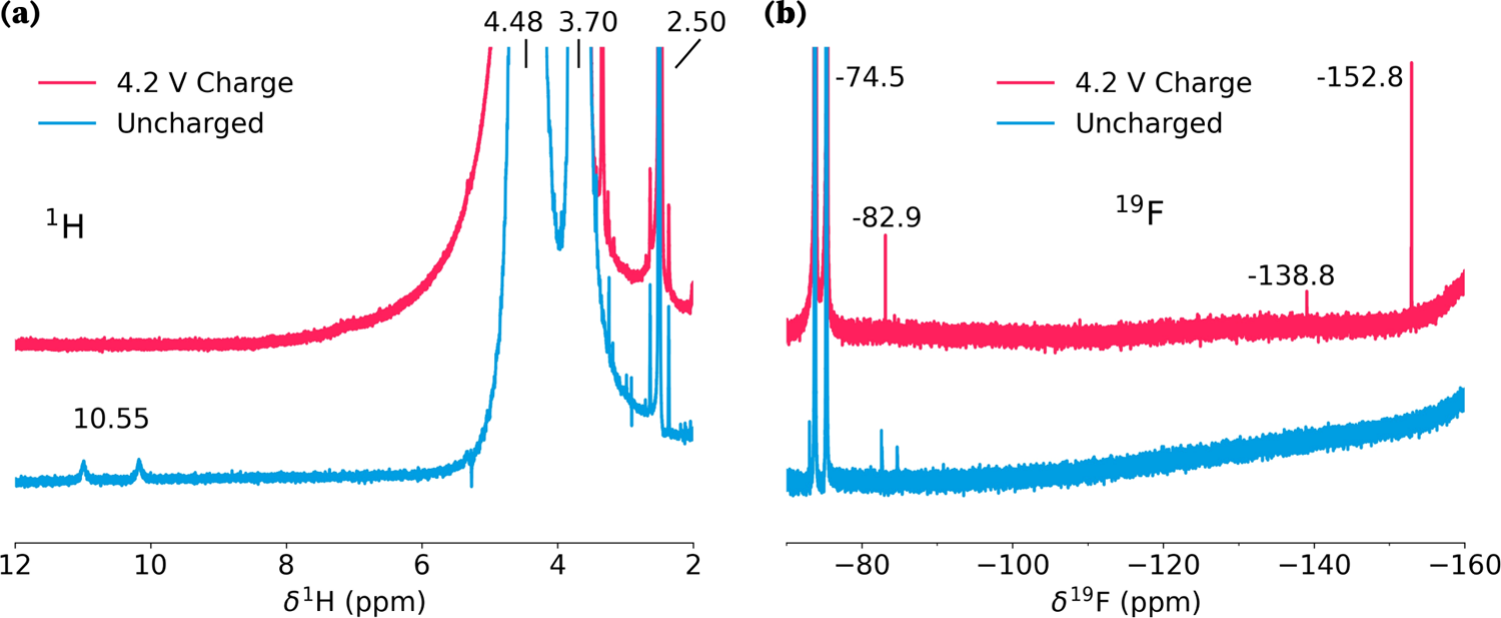
(a) ^1^H NMR spectrum of LP30 pristine electrolyte (blue) and electrolyte extracted from the separator at the end of the first 4.2 V charge (pink) in DMSO–d_6_. The main signals are assigned to EC (4.48 ppm), DMC (3.70 ppm), HF (10.55 ppm), and DMSO (2.50 ppm). (b) Corresponding ^19^F NMR spectrum, here the main signals, are assigned to PF_6_^–^ (−74.5 ppm), oxyfluorophosphate salts (−82.9 ppm), SiF_*x*_(−138.8 ppm), and BF_4_^–^ (−152.8 ppm). Further details on peak assignment can be found in Section S8 in the SI.

To shed further light on the reaction pathway, solution nuclear magnetic resonance (NMR) spectroscopy of the electrolyte extracted from the coin cell separators was carried out after the first charge and compared with a pristine electrolyte. The ^1^H NMR spectra, depicted in Figure 6a, reveal the complete consumption of the HF signal detected in the uncycled electrolyte (10.55 ppm, ^1^*J*(^1^H,^19^F) = 410 Hz). The ^19^F NMR spectrum of the charged electrolyte shows the formation of oxyfluorophosphate salts (−82.9 ppm) associated with the decomposition products of widely reported LiPF_6_ hydrolysis:^48,49^
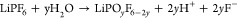
1

It has been shown that the LiPF_6_ hydrolysis reaction is accelerated at high temperatures like the ones used in our study, even by small traces of water.^50^ Such traces of water could be introduced by battery components,^51^ specifically those with a high active surface such as the 30 wt% conductive carbon, or produced by electrolyte degradation.^52^ Thus, we observe consumption of already present HF and traces of its further generation, which could provide H^+^ and fluoride ions to drive the reaction with Fe metal. Additionally, the ^19^F NMR spectra also show traces of SiF_*x*_ and BF_4_^–^ species, byproducts of HF’s interaction with borosilicate glass under conditions of high HF concentration.^53^

We now return to the ^57^Fe Mössbauer spectroscopy results presented in Figure 4. We associate the third Fe(II) signal (green) in the 3 V charged sample with an FeF_2_ phase produced by the Fe impurity reaction. The experimentally determined isomer shift of δ = 1.27(2) mm·s^–1^ is consistent with previous literature reports on *n*-FeF_2_ nanoparticles,^54^ with the quadrupole splitting deviating from previously reported values due to the different morphology of the materials.^55^

**Figure 7:**
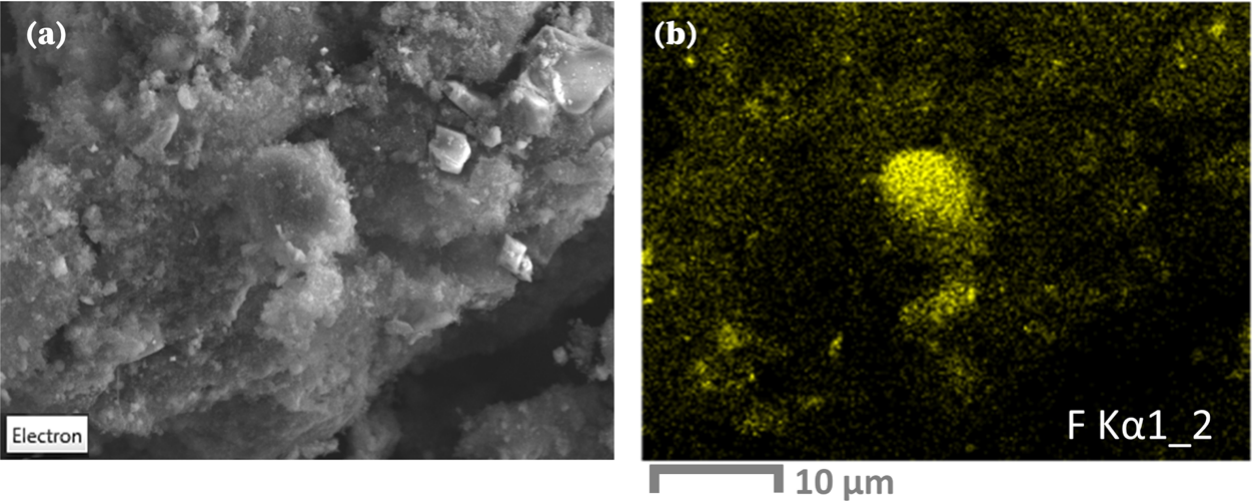
(a) SEM electron image of the cathode material at the end of 4.2 V charge. (b) EDS map of the F Kα signal collected for the charged cathode material.

This hypothesis is corroborated by SEM-EDS maps of the 4.2 V charged cathode, which revealed regions within the porous conductive carbon coated with fluorine, Figure 7. The electron image in Figure 7a illustrates the porous texture of these deposits. These findings align with FeF_2_ deposits observed in the literature for the corrosion of iron in the battery grade electrolyte 1 M LiPF_6_ in ethylene carbonate/diethyl carbonate solvent (EC/DEC = 50/50 v/v).^56^

In light of the above-presented experimental evidence, we suggest a possible irreversible reaction mechanism for the Fe metal impurity. Studies on Fe as a current collector under a nonaqueous alkyl carbonate solution containing LiPF_6_ salt have shown that below 3 V vs Li/Li^+^, the metal surface is covered by a thin protective native oxide layer.^57^ We expect H^+^ generated by the LiPF_6_ hydrolysis to chemically react with the native oxide film, exposing the underlying reactive Fe metal:
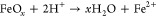
2followed by the direct electrochemical oxidation of Fe metal,
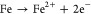
3

The dissolved Fe(II) species could then react in solution with the F^–^ ions from the surrounding electrolyte and precipitate on the cathode, forming the porous deposits observed in the SEM-EDS. Further reactions, studied in the literature for Cu and Al metal current collectors,^58−60^ could be coexistent with the direct oxidation mechanism proposed above:
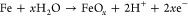
4
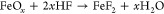
5

The above equations result in a consumption of the Fe metal at the 3 V plateau and the formation of FeF_2_ amorphous porous precipitates on the cathode carbon surface. A recent study by Yu et al.^61^ looks at the performance of Fe metal nanopowders, ball milled with LiF and Li_3_PO_4_, as lithium-salt-composite cathodes for LIBs. The study reports the irreversible conversion of the crystalline Fe metal particles into an amorphous phase of iron salts throughout the first charge. High-resolution tunneling electron microscopy and X-ray absorption spectroscopy are used to confirm Fe is found in Fe(II) and Fe(III) oxidation states at the end of the first charge, in a mixture of amorphous phases of FeF_*x*_ and FePO_4_. The mechanisms identified in this study and the voltage profiles of the capacity, attributed to the oxidation of nano-Fe(0) domains within the amorphous phase of iron salts, are in excellent agreement with the hypothesis of this work.

However, we note the absence of an Fe metal signal in the 4.2 V charge EDS maps of the porous deposits (Figures S4–5) and a lack of a third Fe(II) component in the 4.2 V charged Mössbauer fit (bottom spectra of Figure 4). These results indicate that a further mechanism commences after the 3 V plateau. Previous studies report dissolution of Fe species evolved from Fe(II) surface degradation coatings of Fe metal current collectors in battery electrolytes,^57,62^ and others observed Fe dissolution from battery cathode FeF_2_.^63^ We therefore expect the precipitated high surface area Fe species to further dissolve into the electrolyte, as reported for FeF_2_ cathodes for LIBs.^64,65^ These species are expected to deposit on the anode, as explored by Ruff et al.^66^ Inductively coupled plasma (ICP) analysis of the Li metal anodes was performed, yielding inconclusive results; see Section S9 of the SI.

**Figure 8:**
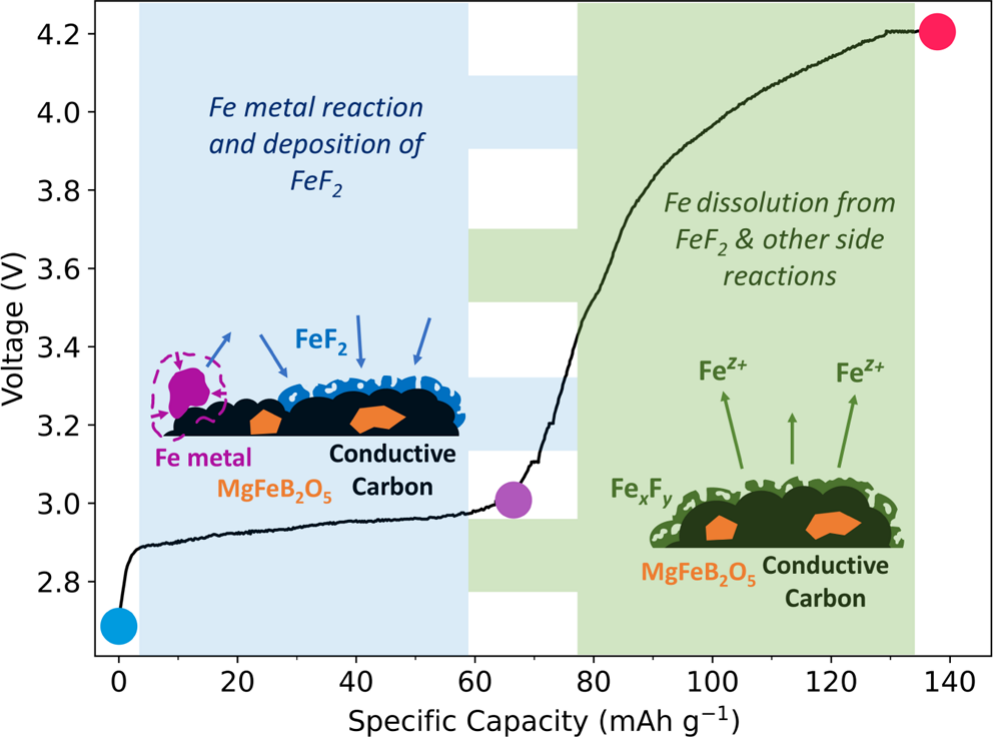
Graphical illustration of the proposed reaction pathway, superposed on the electrochemical data of our cathode material against Li metal. The enlarged dots correspond to the three main cycling points at which postcycling analysis was collected: uncharged (blue), post 3 V plateau (purple), and end of charge at 4.2 V (pink).

Our proposed reaction pathway is summarized in Figure 8. We suggest that the plateau in the first charge capacity observed in our material can be attributed to an electrochemical reaction of the Fe metal impurity initiated by HF within the electrolyte, resulting in the precipitation of amorphous FeF_2_ deposits on the cathode. We further hypothesize the dissolution of Fe species from the high surface area FeF_2_ deposits at voltages above 3 V, which likely deposit on the Li metal anode. The capacity generated by the cell above the 3 V plateau could be ascribed to a combination of side reactions in the electrolyte, degradation of cell components at the high cycling temperature, reactions with the high surface area carbon used as a conductive additive, and the degradation of an amorphous surface coating of the polyanion material. The details of this higher voltage mechanism will be elucidated in a future manuscript.

## Conclusions

This work provides a comprehensive study of the electrochemical performance of cathode candidate MgFeB_2_O_5_. Electrochemical tests revealed a near-theoretical first charge capacity against a lithium metal anode, initially suggesting that MgFeB_2_O_5_ is a promising cathode candidate. However, we present experimental verification of the lack of Mg mobility within the structure, as well as an effort to determine the origin of the capacity reached at first charge through a wide range of postcycling characterization techniques. We propose a reaction mechanism involving the irreversible electrochemical reaction of a small wt% Fe metal impurity present in the main cathode phase, initiated by HF in the electrolyte.

In the literature, proof of Mg ion reversible insertion–deinsertion relies too strongly on measured capacity, or at most structural changes, rather than on detailed structural and chemical analysis.^18^ This study is a concrete example of similar behaviors being induced by competing reactions with trace impurities or within the electrolyte. Our work underscores the complexity of cathode material behavior in rechargeable Mg batteries and highlights the critical role of comprehensive postcycling characterization in unraveling these mechanisms and understanding Mg deintercalation.

## Experimental Section

MgFeB_2_O_5_ was synthesized via a solid-state synthesis method. Stoichiometric amounts of FeC_2_O_4_· *x* H_2_O (*x* ≈ 2, Alfa Aesar 96%), Mg(CH_3_CO_2_)_2_·4H_2_O (Alfa Aesar 98%), and H_3_BO_3_ (Alfa Aesar 99.99%) were ball milled for 25 min. The mixture was heated to 400 °C at 3 °C min^–1^ and held for 10 h under flowing Ar in a Al_2_O_3_ crucible wrapped in Cu foil to reduce oxidation. The heated reagents were then reground in a planetary ball mill for 25 min to obtain a fine powder. This powder was pressed into a 13 mm diameter pellet and subsequently heated in an Al_2_O_3_ crucible wrapped in Cu foil to 950 °C at 3 °C min^–1^, held at this temperature for 10 h, and furnace cooled, under flowing argon.

Coin cells for electrochemical testing were prepared by mixing 60 wt% hand-ground MgMnB_2_O_5_ powder with 30 wt% Ketjen Black Carbon and 10 wt% binder: PVDF (Kynar Flex 2801-00). Stainless steel CR2032 cells were assembled with ≈6 mg of loose cathode powder, combined with a Whatman glass fiber separator (GF/B 55 mm diameter) soaked in LP30 electrolyte (Sigma-Aldrich) (1 M LiPF_6_ in 50/50 v/v ethylene carbonate and dimethyl carbonate, EC/DMC) vs Li metal anode. Electrochemical tests were performed using galvanostatic charge/discharge on a Landt battery tester between 2.8 and 4.2 V in an oven at 55 °C. The *ex situ* cathode samples were disassembled in an Ar atmosphere glovebox and washed in DMC to remove residue electrolytes and salts. *Ex situ* scanning electron microscopy energy-dispersive X-ray spectroscopy (SEM-EDS) maps and spectra were acquired using an Oxford Instruments X-maxN 80 EDS system on a TESCAN MIRA3 FEG-SEM operated at 10 kV.

Synchrotron powder X-ray diffraction (SXRD) was collected on samples at the Diamond Light Source I11 beamline (λ = 0.824110 Å) using the Mythen-2 position-sensitive detector at room temperature. Samples were packed into a 0.3 mm diameter capillary and sealed with Araldite adhesive bicomponent epoxy under a controlled Ar atmosphere. TOPAS^67^ was used to perform Rietveld refinement^68^ on the resulting SXRD data over a 2θ range of 10–80°. The peak shapes for Rietveld analysis were modeled using a pseudo-Voigt function^69^ and the background modeled using a Chebyshev polynomial with 17 terms.

*Ex situ*^57^Fe Mössbauer spectroscopy was conducted at the Institute Charles Gherard Montpellier on a lab-based Mössbauer spectrometer with a ^57^Co source. Spectra were collected on 3–5 mg of cathodes harvested from coin cells cycled in Cambridge. Owing to the limited quantity of cathode material retrievable from each coin cell, the Mössbauer spectra collection extended over a two-day period to collect a satisfactory signal-to-noise ratio for analysis.

Fe K-edge X-ray absorption near edge spectroscopy (XANES) spectra were collected at the B18 beamline^70,71^ of Diamond Light Source using customized *operando* cells with MgFeB_2_O_5_ cathodes vs Li metal. An X-ray beam was vertically collimated using a Pt-coated mirror before passing through the Si(111) double crystal monochromator. High-order harmonics in an incident beam were eliminated by using two dedicated Pt-coated mirrors operating at an 8 mrad incidence angle. Energy calibration was achieved by the simultaneous measurement of a 5 μm thick Fe metal foil using Athena Software.^72^ Cells were cycled at a rate of C/25 at ambient temperature, and XANES spectra were intermittently recorded at 10 min intervals in transmission mode using the quick extended X-ray absorption fine structure technique. The room-temperature *operando* cycling reproduced the electrochemical behavior observed in our laboratory-based coin cells, albeit reaching a reduced capacity than that achieved at 55 °C, a phenomenon we attribute to the slower kinetics at lower operational temperatures, as seen in Figure S6 in the SI.

A Quantum Design magnetic properties measurement system (MPMS3) superconducting quantum interference device (SQUID) magnetometer was used for magnetic measurements. Cathode materials were extracted from charged coin cells in an inert Ar glovebox and packed in a brass sample holder. Magnetic hysteresis curves were recorded at 200 K by recording sample magnetization (M) as a function of an externally applied field ranging from 0 to 7 T.

Samples for nuclear magnetic resonance (NMR) measurements were prepared by mixing pristine LP30 electrolyte with a small amount of deuterated dimethyl sulfoxide (DMSO-d_6_) or by extracting the electrolyte from the separator of a cycled cell by soaking it in a vial of DMSO-d_6_. The samples were then placed into 5 mm NMR tubes fitted with J-Young taps. The NMR experiments were performed on a Bruker Avance IIIHD spectrometer equipped with an 11.7 T magnet (ν_0_(^1^H) = 500.20 MHz and ν_0_(^19^F) = 470.60 MHz) and a BBO probe. Spectra were acquired by using a Bloch decay pulse sequence with optimized pulse lengths. ^1^H chemical shifts were referenced to the residual proton signal of the DMSO-d_6_ solvent (δ_iso_ = 2.50 ppm), and ^19^F shifts were referenced to PF_6_^–^ (δ_iso_ = −74.5 ppm).

## Supporting Information Available

Tables with refined Rietveld parameters for MgFeB_2_O_5_ and Fe metal impurity; full SEM-EDS element maps and tables for images presented in the paper; and electrochemistry curve of *operando* XANES cell. Full discussion of Mössbauer spectra, including tables and individual fit images. Explanation for (i) conversion of Mössbauer at% to wt% for SXRD comparison and (ii) calculation of capacity expected from Fe impurity wt%. Discussion on the irreversibility of Fe metal impurity through first cycle discharge and XANES data. A full NMR quantification of spectra, Karl Fischer titration of electrolyte, and ICP results of Li metal anodes (PDF)

## References

[ref1] Weitemeyer S.; Kleinhans D.; Vogt T.; Agert C. Integration of Sources in future power systems: The role of storage. Renewable Energy 2015, 75, 14–20. 10.1016/j.renene.2014.09.028.

[ref2] Blomgren G. E. The Development and Future of Lithium Ion Batteries. J. Electrochem. Soc. 2017, 164, A5019–5052. 10.1149/2.0251701jes.

[ref3] Schmuch R.; Wagner R.; Hörpel G.; Placke T.; Winter M. Performance and cost of materials for lithium-based rechargeable automotive batteries. Nat. Energy 2018, 3, 267–278. 10.1038/s41560-018-0107-2.

[ref4] Ryu H.-H.; Sun H. H.; Myung S.-T.; Yoon C. S.; Sun Y.-K. Reducing cobalt from lithium-ion batteries for the electric vehicle era. Energy Environ. Sci. 2021, 14, 844–852. 10.1039/D0EE03581E.

[ref5] Doughty D. H.; Roth E. P. A General Discussion of Li Ion Battery Safety. Electrochem. Soc. Interface 2012, 21, 37 10.1149/2.f03122if.

[ref6] Escobar-Hernandez H. U.; Gustafson R. M.; Papadaki M. I.; Sachdeva S.; Mannan M. S. Thermal Runaway in Lithium-Ion Batteries: Incidents, Kinetics of the Runaway and Assessment of Factors Affecting Its Initiation. J. Electrochem. Soc. 2016, 163, A2691 10.1149/2.0921613jes.

[ref7] Chen Y.; Kang Y.; Zhao Y.; Wang L.; Liu J.; Li Y.; Liang Z.; He X.; Li X.; Tavajohi N.; Li B. A review of lithium-ion battery safety concerns: The issues, strategies, and testing standards. J. Energy Chem. 2021, 59, 83–99. 10.1016/j.jechem.2020.10.017.

[ref8] Walter M.; Kovalenko M. V.; Kravchyk K. V. Challenges and benefits of post-lithium-ion batteries. New J. Chem. 2020, 44, 1677–1683. 10.1039/C9NJ05682C.

[ref9] Liang Y.; Dong H.; Aurbach D.; Yao Y. Current status and future directions of multivalent metal-ion batteries. Nat. Energy 2020, 5, 646–656. 10.1038/s41560-020-0655-0.

[ref10] Canepa P.; Sai Gautam G.; Hannah D. C.; Malik R.; Liu M.; Gallagher K. G.; Persson K. A.; Ceder G. Odyssey of Multivalent Cathode Materials: Open Questions and Future Challenges. Chem. Rev. 2017, 117, 4287–4341. 10.1021/acs.chemrev.6b00614. 28269988

[ref11] Bitenc J.; Dominko R. Opportunities and Challenges in the Development of Cathode Materials for Rechargeable Mg Batteries. Front. Chem. 2018, 6, 1–10. 10.3389/fchem.2018.00634. 30619838 PMC6305455

[ref12] Levi E.; Gofer Y.; Vestfreed Y.; Lancry E.; Aurbach D. Cu_2_Mo_6_S_8_ Chevrel Phase, A Promising Cathode Material for New Rechargeable Mg Batteries: A Mechanically Induced Chemical Reaction. Chem. Mater. 2002, 14, 2767–2773. 10.1021/cm021122o.

[ref13] Aurbach D.; Lu Z.; Schechter A.; Gofer Y.; Gizbar H.; Turgeman R.; Cohen Y.; Moshkovich M.; Levi E. Prototype Systems for Rechargeable Magnesium Batteries. Nature 2000, 407, 724–727. 10.1038/35037553. 11048714

[ref14] Lancry E.; Levi E.; Gofer Y.; Levi M.; Salitra G.; Aurbach D. Leaching Chemistry and the Performance of the Mo_6_S_8_ Cathodes in Rechargeable Mg Batteries. Chem. Mater. 2004, 16, 2832–2838. 10.1021/cm034944+.

[ref15] Wan L. F.; Perdue B. R.; Apblett C. A.; Prendergast D. Mg Desolvation and Intercalation Mechanism at the Mo_6_S_8_ Chevrel Phase Surface. Chem. Mater. 2015, 27, 5932–5940. 10.1021/acs.chemmater.5b01907.

[ref16] Pei C.; Xiong F.; Yin Y.; Liu Z.; Tang H.; Sun R.; An Q.; Mai L. Recent Progress and Challenges in the Optimization of Electrode Materials for Rechargeable Magnesium Batteries. Small 2021, 17, 2004108 10.1002/smll.202004108. 33354934

[ref17] Medina A.; Pérez-Vicente C.; Alcántara R. Advancing towards a Practical Magnesium Ion Battery. Materials 2021, 14, 7488 10.3390/ma14237488. 34885643 PMC8659073

[ref18] Johnson I. D.; Ingram B. J.; Cabana J. The Quest for Functional Oxide Cathodes for Magnesium Batteries: A Critical Perspective. ACS Energy Lett. 2021, 6, 1892–1900. 10.1021/acsenergylett.1c00416.

[ref19] Mao M.; Gao T.; Hou S.; Wang C. A critical review of cathodes for rechargeable Mg batteries. Chem. Soc. Rev. 2018, 47, 8804–8841. 10.1039/C8CS00319J. 30339171

[ref20] Li Z.; Häcker J.; Fichtner M.; Zhao-Karger Z. Cathode Materials and Chemistries for Magnesium Batteries: Challenges and Opportunities. Adv. Energy Mater. 2023, 13, 2300682 10.1002/aenm.202300682.

[ref21] Dey S.; Lee J.; Britto S.; Stratford J. M.; Keyzer E. N.; Dunstan M. T.; Cibin G.; Cassidy S. J.; Elgaml M.; Grey C. P. Exploring Cation-Anion Redox Processes in One-Dimensional Linear Chain Vanadium Tetrasulfide Rechargeable Magnesium Ion Cathodes. J. Am. Chem. Soc. 2020, 142, 19588–19601. 10.1021/jacs.0c08222. 33108185

[ref22] Bayliss R. D.; Key B.; Sai Gautam G.; Canepa P.; Kwon B. J.; Lapidus S. H.; Dogan F.; Adil A. A.; Lipton A. S.; Baker P. J.; Ceder G.; Vaughey J. T.; Cabana J. Probing Mg Migration in Spinel Oxides. Chem. Mater. 2020, 32, 663–670. 10.1021/acs.chemmater.9b02450.

[ref23] Okamoto S.; Ichitsubo T.; Kawaguchi T.; Kumagai Y.; Oba F.; Yagi S.; Shimokawa K.; Goto N.; Doi T.; Matsubara E. Intercalation and Push-Out Process with Spinel-to-Rocksalt Transition on Mg Insertion into Spinel Oxides in Magnesium Batteries. Adv. Sci. 2015, 2, 1500072 10.1002/advs.201500072. PMC5115418 27980965

[ref24] Muldoon J.; Bucur C. B.; Gregory T. Fervent Hype behind Magnesium Batteries: An Open Call to Synthetic Chemists–Electrolytes and Cathodes Needed. Angew. Chem., Int. Ed. 2017, 56, 12064–12084. 10.1002/anie.201700673. 28295967

[ref25] Ling J.; Karuppiah C.; Krishnan S. G.; Reddy M. V.; Misnon I. I.; Ab Rahim M. H.; Yang C.-C.; Jose R. Phosphate Polyanion Materials as High-Voltage Lithium-Ion Battery Cathode: A Review. Energy Fuels 2021, 35, 10428–10450. 10.1021/acs.energyfuels.1c01102.

[ref26] Huang H.; Yin S.-C.; Nazar L. F. Approaching Theoretical Capacity of LiFePO_4_ at Room Temperature at High Rates. Electrochem. Solid-State Lett. 2001, 4, A170 10.1149/1.1396695.

[ref27] Huang Z.-D.; Masese T.; Orikasa Y.; Mori T.; Minato T.; Tassel C.; Kobayashi Y.; Kageyama H.; Uchimoto Y. MgFePO_4_F as a feasible cathode material for magnesium batteries. J. Mater. Chem. A 2014, 2, 11578–11582. 10.1039/C4TA01779J.

[ref28] Cabello M.; Alcántara R.; Nacimiento F.; Lavela P.; Aragón M. J.; Tirado J. L. Na_3_V_2_(PO_4_)_3_ as electrode material for rechargeable magnesium batteries: a case of sodium-magnesium hybrid battery. Electrochim. Acta 2017, 246, 908–913. 10.1016/j.electacta.2017.06.080.

[ref29] Ling C.; Banerjee D.; Song W.; Zhang M.; Matsui M. First-principles study of the magnesiation of olivines: redox reaction mechanism, electrochemical and thermodynamic properties. J. Mater. Chem. 2012, 22, 13517–13523. 10.1039/c2jm31122d.

[ref30] Yang S.-H.; Xue H.; Guo S.-P. Borates as promising electrode materials for rechargeable batteries. Coord. Chem. Rev. 2021, 427, 213551 10.1016/j.ccr.2020.213551.

[ref31] Sanglay G. D. D.; Garcia J. S.; Palaganas M. S.; Sorolla M.; See S.; Limjuco L. A.; Ocon J. D. Borate-Based Compounds as Mixed Polyanion Cathode Materials for Advanced Batteries. Molecules 2022, 27 (22), 8047 10.3390/molecules27228047. 36432146 PMC9695605

[ref32] Ni Q.; Bai Y.; Wu F.; Wu C. Polyanion-Type Electrode Materials for Sodium-Ion Batteries. Adv. Sci. 2017, 4, 1600275 10.1002/advs.201600275. PMC5357992 28331782

[ref33] Bo S. H.; Grey C. P.; Khalifah P. G. Defect-Tolerant Diffusion Channels for Mg^2+^ Ions in Ribbon-Type Borates: Structural Insights into Potential Battery Cathodes MgVBO_4_ and Mg_x_Fe_2–x_B_2_O_5_. Chem. Mater. 2015, 27, 4630–4639. 10.1021/acs.chemmater.5b01040.

[ref34] Glass H. F. J.; Liu Z.; Bayley P. M.; Suard E.; Bo S.-H.; Khalifah P. G.; Grey C. P.; Dutton S. E. Mg_x_Mn_2–x_B_2_O_5_ Pyroborates (2/3 ⩽) x ⩽ 4/3): High Capacity and High Rate Cathodes for Li-Ion Batteries. Chem. Mater. 2017, 29, 3118–3125. 10.1021/acs.chemmater.7b00177.

[ref35] Gütlich P. Fifty Years of Mossbauer Spectroscopy in Solid State Research—Remarkable Achievements, Future Perspectives. Z. Anorg. Allg. Chem. 2012, 638, 15–43. 10.1002/zaac.201100416.

[ref36] Shinno I.; Zhe L. Octahedral site Fe^2+^ quadrupole splitting distributions from the Moessbauer spectra of arrojadite. Am. Mineral. 1998, 83, 1316–1322. 10.2138/am-1998-11-1220.

[ref37] Ingalls R. Electric-Field Gradient Tensor in Ferrous Compounds. Phys. Rev. 1964, 133, A787–A795. 10.1103/PhysRev.133.A787.

[ref38] Lipson A. L.; Han S.-D.; Pan B.; See K. A.; Gewirth A. A.; Liao C.; Vaughey J. T.; Ingram B. J. Practical Stability Limits of Magnesium Electrolytes. J. Electrochem. Soc. 2016, 163, A2253 10.1149/2.0451610jes.

[ref39] Deivanayagam R.; Ingram B. J.; Shahbazian-Yassar R. Progress in development of electrolytes for magnesium batteries. Energy Storage Mater. 2019, 21, 136–153. 10.1016/j.ensm.2019.05.028.

[ref40] Saha P.; Datta M. K.; Velikokhatnyi O. I.; Manivannan A.; Alman D.; Kumta P. N. Rechargeable magnesium battery: Current status and key challenges for the future. Prog. Mater. Sci. 2014, 66, 1–86. 10.1016/j.pmatsci.2014.04.001.

[ref41] Bella F.; De Luca S.; Fagiolari L.; Versaci D.; Amici J.; Francia C.; Bodoardo S. An Overview on Anodes for Magnesium Batteries: Challenges towards a Promising Storage Solution for Renewables. Nanomaterials 2021, 11 (3), 810 10.3390/nano11030810. 33809914 PMC8004101

[ref42] Wang J.; Chen-Wiegart Y. K.; Wang J. In operando tracking phase transformation evolution of lithium iron phosphate with hard X-ray microscopy. Nat. Commun. 2014, 5, 4570 10.1038/ncomms5570. 25087693

[ref43] Gong Z.; Yang Y. Recent advances in the research of polyanion-type cathode materials for Li-ion batteries. Energy Environ. Sci. 2011, 4, 3223–3242. 10.1039/c0ee00713g.

[ref44] Padhi A. K.; Nanjundaswamy K. S.; Goodenough J. B. Phospho-olivines as Positive-Electrode Materials for Rechargeable Lithium Batteries. J. Electrochem. Soc. 1997, 144, 1188–1194. 10.1149/1.1837571.

[ref45] Chen G.; Wilcox J. D.; Richardson T. J. Improving the Performance of Lithium Manganese Phosphate Through Divalent Cation Substitution. Electrochem. Solid-State Lett. 2008, 11, A190 10.1149/1.2971169.

[ref46] Devi E. C.; Singh S. D. Tracing the Magnetization Curves: a Review on Their Importance, Strategy, and Outcomes. J. Supercond. Novel Magn. 2021, 34, 15–25. 10.1007/s10948-020-05733-6.

[ref47] Iwakura C.; Fukumoto Y.; Inoue H.; Ohashi S.; Kobayashi S.; Tada H.; Abe M. Electrochemical characterization of various metal foils as a current collector of positive electrode for rechargeable lithium batteries. J. Power Sources 1997, 68, 301–303. 10.1016/S0378-7753(97)02538-X.

[ref48] Stich M.; Göttlinger M.; Kurniawan M.; Schmidt U.; Bund A. Hydrolysis of LiPF_6_ in Carbonate-Based Electrolytes for Lithium-Ion Batteries and in Aqueous Media. J. Phys. Chem. C 2018, 122, 8836–8842. 10.1021/acs.jpcc.8b02080.

[ref49] Liu M.; Vatamanu J.; Chen X.; Xing L.; Xu K.; Li W. Hydrolysis of LiPF_6_-Containing Electrolyte at High Voltage. ACS Energy Lett. 2021, 6, 2096–2102. 10.1021/acsenergylett.1c00707.

[ref50] Wiemers-Meyer S.; Winter M.; Nowak S. Mechanistic insights into lithium ion battery electrolyte degradation—a quantitative NMR study. Phys. Chem. Chem. Phys. 2016, 18, 26595–26601. 10.1039/C6CP05276B. 27711648

[ref51] Huttner F.; Haselrieder W.; Kwade A. The Influence of Different Post-Drying Procedures on Remaining Water Content and Physical and Electrochemical Properties of Lithium-Ion Batteries. Energy Technol. 2020, 8, 1900245 10.1002/ente.201900245.

[ref52] Rinkel B. L. D.; Hall D. S.; Temprano I.; Grey C. P. Electrolyte Oxidation Pathways in Lithium-Ion Batteries. J. Am. Chem. Soc. 2020, 142, 15058–15074. 10.1021/jacs.0c06363. 32697590

[ref53] Rinkel B. L. D.; Hall D. S.; Temprano I.; Grey C. P. Electrolyte Oxidation Pathways in Lithium-Ion Batteries. J. Am. Chem. Soc. 2020, 142, 15058–15074. 10.1021/jacs.0c06363. 32697590

[ref54] Conte D. E.; Di Carlo L.; Sougrati M. T.; Fraisse B.; Stievano L.; Pinna N. Operando Mössbauer Spectroscopy Investigation of the Electrochemical Reaction with Lithium in Bronze-Type FeF_3_·0.33H_2_O. J. Phys. Chem. C 2016, 120, 23933–23943. 10.1021/acs.jpcc.6b06711.

[ref55] Ramasamy S.; Jiang J.; Gleiter H.; Birringer R.; Gonser U. Investigation of nanocrystalline FeF_2_ by Mössbauer spectroscopy. Solid State Commun. 1990, 74, 851–855. 10.1016/0038-1098(90)90949-C.

[ref56] Guitián B.; Nóvoa X.; Pintos A. Development of conversion coatings on iron via corrosion in LiPF_6_ solution. Electrochim. Acta 2019, 304, 428–436. 10.1016/j.electacta.2019.03.011.

[ref57] Myung S.-T.; Sasaki Y.; Sakurada S.; Sun Y.-K.; Yashiro H. Electrochemical behavior of current collectors for lithium batteries in non-aqueous alkyl carbonate solution and surface analysis by ToF-SIMS. Electrochim. Acta 2009, 55, 288–297. 10.1016/j.electacta.2009.08.051.

[ref58] Shu J.; Shui M.; Huang F.; Xu D.; Ren Y.; Hou L.; Cui J.; Xu J. Comparative study on surface behaviors of copper current collector in electrolyte for lithium-ion batteries. Electrochim. Acta 2011, 56, 3006–3014. 10.1016/j.electacta.2011.01.004.

[ref59] Zhu P.; Gastol D.; Marshall J.; Sommerville R.; Goodship V.; Kendrick E. A review of current collectors for lithium-ion batteries. J. Power Sources 2021, 485, 229321 10.1016/j.jpowsour.2020.229321.

[ref60] Guo L.; Thornton D. B.; Koronfel M. A.; Stephens I. E. L.; Ryan M. P. Degradation in lithium ion battery current collectors. J. Phys.: Energy 2021, 3, 032015 10.1088/2515-7655/ac0c04.

[ref61] Yu M.; Wang J.; Lei M.; Jung M. S.; Zhuo Z.; Yang Y.; Zheng X.; Sandstrom S.; Wang C.; Yang W.; Jiang D.; Liu T.; Ji X. Unlocking iron metal as a cathode for sustainable Li-ion batteries by an anion solid solution. Sci. Adv. 2024, 10, eadn4441 10.1126/sciadv.adn4441. 38781334 PMC11114228

[ref62] Löchel B.; Strehblow H.-H. Breakdown of passivity of iron by fluoride. Electrochim. Acta 1983, 28, 565–571. 10.1016/0013-4686(83)85043-9.

[ref63] Huang Q.; Turcheniuk K.; Ren X.; Magasinski A.; Gordon D.; Bensalah N.; Yushin G. Insights into the Effects of Electrolyte Composition on the Performance and Stability of FeF_2_ Conversion-Type Cathodes. Adv. Energy Mater. 2019, 9, 1803323 10.1002/aenm.201803323.

[ref64] Gu W.; Magasinski A.; Zdyrko B.; Yushin G. Metal Fluorides Nanoconfined in Carbon Nanopores as Reversible High Capacity Cathodes for Li and Li-Ion Rechargeable Batteries: FeF_2_ as an Example. Adv. Energy Mater. 2015, 5, 1401148 10.1002/aenm.201500243.

[ref65] Gu W.; Borodin O.; Zdyrko B.; Lin H.-T.; Kim H.; Nitta N.; Huang J.; Magasinski A.; Milicev Z.; Berdichevsky G.; Yushin G. Lithium-Iron Fluoride Battery with In Situ Surface Protection. Adv. Funct. Mater. 2016, 26, 1507–1516. 10.1002/adfm.201504848.

[ref66] Ruff Z.; Xu C.; Grey C. P. Transition Metal Dissolution and Degradation in NMC811-Graphite Electrochemical Cells. J. Electrochem. Soc. 2021, 168, 060518 10.1149/1945-7111/ac0359.

[ref67] Coelho A. A. TOPAS and TOPAS-Academic: an optimization program integrating computer algebra and crystallographic objects written in C++. J. Appl. Crystallogr. 2018, 51, 210–218. 10.1107/S1600576718000183.

[ref68] Rietveld H. M. A profile refinement method for nuclear and magnetic structures. J. Appl. Crystallogr. 1969, 2, 65–71. 10.1107/S0021889869006558.

[ref69] Young R. A.; Wiles D. B. Profile shape functions in Rietveld refinements. J. Appl. Crystallogr. 1982, 15, 430–438. 10.1107/S002188988201231X.

[ref70] Diaz-Moreno S.; Amboage M.; Basham M.; et al. The Spectroscopy Village at Diamond Light Source. J. Synchrotron Radiat. 2018, 25, 998–1009. 10.1107/S1600577518006173. 29979161 PMC6038600

[ref71] Dent A. J.; Cibin G.; Ramos S.; Parry S. A.; Gianolio D.; Smith A. D.; Scott S. M.; Varandas L.; Patel S.; Pearson M. R.; Hudson L.; Krumpa N. A.; Marsch A. S.; Robbins P. E. Performance of B18, the Core EXAFS Bending Magnet beamline at Diamond. J. Phys. 2013, 430, 012023 10.1088/1742-6596/430/1/012023.

[ref72] Ravel B.; Newville M. ATHENA and ARTEMIS: interactive graphical data analysis using IFEFFIT. Phys. Scr. 2005, 2005, 1007 10.1238/Physica.Topical.115a01007. 15968136

