# Supplementary material for: Role of Fe Impurity Reactions in the Electrochemical Properties of MgFeB2O5
Source: Chem Mater. 2024 Dec 16;37(1):463–72. doi: 10.1021/acs.chemmater.4c02855 (PMC11736679; doi:10.1021/acs.chemmater.4c02855)
Supplement: Supplementary file 1 — cm4c02855_si_001.pdf [file cm4c02855_si_001.pdf]

The Role of Fe Impurity Reactions in the Electrochemical Properties of $MgFeB_2O_5$

Camilla Tacconis[1,*] **Sunita Dey**,[2,3], **Carson D. McLaughlin**[1], **Moulay Tahar Sougrati**[4], **Christopher A. O'Keefe**[3], **Iuliia Mikulska**[5], **Clare P. Grey**[3], **Siân E. Dutton**[1,†]

[1] *University of Cambridge, Department of Physics, JJ Thomson Ave, Cambridge, CB3 0HE, UK*
[2] *University of Aberdeen, CAdvanced Centre for Energy and Sustainability (ACES), Department of Chemistry, Aberdeen, hemistry Meston building, Department of Natural and Computational Sciences, Aberdeen, AB24 3FX, UK*
[3] *University of Cambridge, Department of Chemistry Lensfield Road, Cambridge, CB2 1EW, UK*
[4] *Universite de Montpellier, Institut Charles Gerhardt (UMR 5253) CC004, Place Eugene Bataillon, Montpellier, Cedex 5, FR 34095, France*
[5] *Diamond Light Source Ltd, Science Division, Harwell Science & Innovation Campus, Didcot, Oxfordshire OX11 0DE*
[*] *ct603@cam.ac.uk*
[†] *sed33@cam.ac.uk*

Supporting Information

Contents

S1 Synchrotron Powder X-Ray Diffraction 2

S2 SEM EDS 3

S3 *Operando* XANES Electrochemistry 7

S4 Mössbauer Spectra 8

S5 Conversion of Mössbauer At% for SPXRD comparison 10

S6 Calculation of expected Capacity contribution from Fe metal wt % 10

S7 On the Irreversibility of the Fe metal reaction 11

S8 NMR 12

S9 ICP of Li metal Anode 14

S1. Synchrotron Powder X-Ray Diffraction

Table S1. Refined parameters for pristine and cycled $MgFeB_2O_5$ cathode samples obtained through Rietveld refinement of SPXRD data.

Parameters		Pristine	3 V Charge	4.2 V Charge
a (Å)		3.17194(3)	3.17254(5)	3.17011(2)
b (Å)		6.16624(6)	6.16817(8)	6.16337(3)
c (Å)		9.29297(14)	9.2954(2)	9.28706(9)
Volume (Å^3)		175.851(4)	175.998(5)	175.555(2)
χ^2		4.09	2.48	1.97
R_{wp} %		3.10	1.87	1.46
**Cathode** wt %		92.57(2)	96.44(8)	100
**Fe metal** wt %		7.43(2)	3.56(8)	0
**M1**	Mg Occ	0.498(5)	0.494(11)	0.48(23)
	Fe Occ	0.502(5)	0.506(11)	0.51(10)
**M2**	Mg Occ	0.513(5)	0.508(9)	0.48(27)
	Fe Occ	0.487(5)	0.492(9)	0.52(11)

Table S2. Refined SPXRD parameters for Fe metal impurity identified in pristine and cycled $MgFeB_2O_5$ cathode samples. This was identified to have the Cubic "I m -3 m" space group.

Parameters	Pristine	3 V Charge	4.2 V Charge
a,b,c (Å)	2.86812(3)	2.86880(8)	/
Volume (Å^3)	23.594(1)	23.610(2)	/
**Fe Metal** wt %	7.43(2)	3.56(8)	0

S2. SEM EDS

This section contains the detailed results of the SEM EDS images presented in the main paper in Figures 3 and 7.

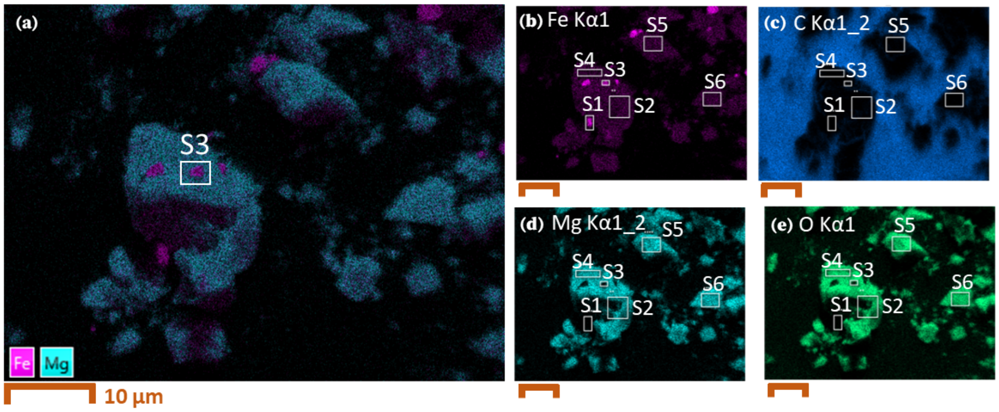

Fig. S1. a) SEM EDS map of $MgFeB_2O_5$ **pristine** cathode material (Fig. 3 of the main paper) with the corresponding elemental mapping images of b) iron, c) carbon d) magnesium and e) oxygen. The Elemental composition obtained from EDS of the various sub-spectra can be found in Table 1 below.

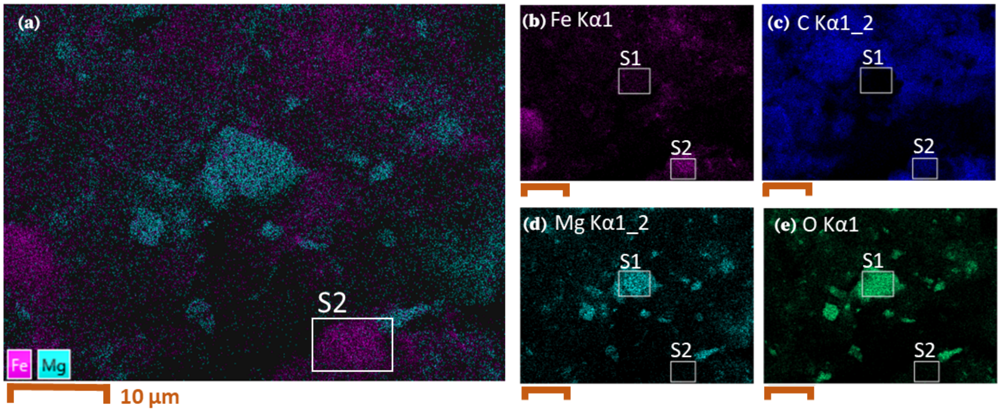

Fig. S2. SEM EDS spectra images of $MgFeB_2O_5$ cathode material **charged to 4.2 V** in a coin cell, (Fig. 3 of the main paper) with the corresponding elemental mapping images of b) iron, c) carbon d) magnesium and e) oxygen. The Elemental composition obtained from EDS of the various sub-spectra can be found in Table 1 below.

Table S3. Relevant At% composition of Spectra as obtained by EDS spectral analysis of the $MgFeB_2O_5$ cathode material samples shown above in Figures S1 and S2 (Seen in Fig. 3 of main paper). The **Pristine** results show that the overall Map spectrum has the expected Mg:Fe ratio, here we also highlight the sub-spectrum S3, which is Fe rich, indicative of the Fe metal impurity. The **Charged** results clearly show that the purple-looking Fe clouds in S2 are an artefact of the binning of the colour map. The sub-spectrum S2 which appears to contain Fe in the elemental mapping image (in Fig. S2 and S4) is solely composed of F, as evidenced in Figure S4 below.

Sample	Spectrum	Fe At%	Mg At%	F At%
**Pristine**	Map Spectrum	1.7	1.6	/
(Fig S1)	S1	92.1	/	/
	S2	10.4	11.6	/
	S3	27.5	9.5	/
	S4	5.0	9.0	/
	S5	4.9	8.5	/
	S6	5.7	10.8	/
**Charge**	Map Spectrum	1.5	1.6	8.1
(Fig S2)	S1	6.5	8.4	/
	S2	/	/	28.2

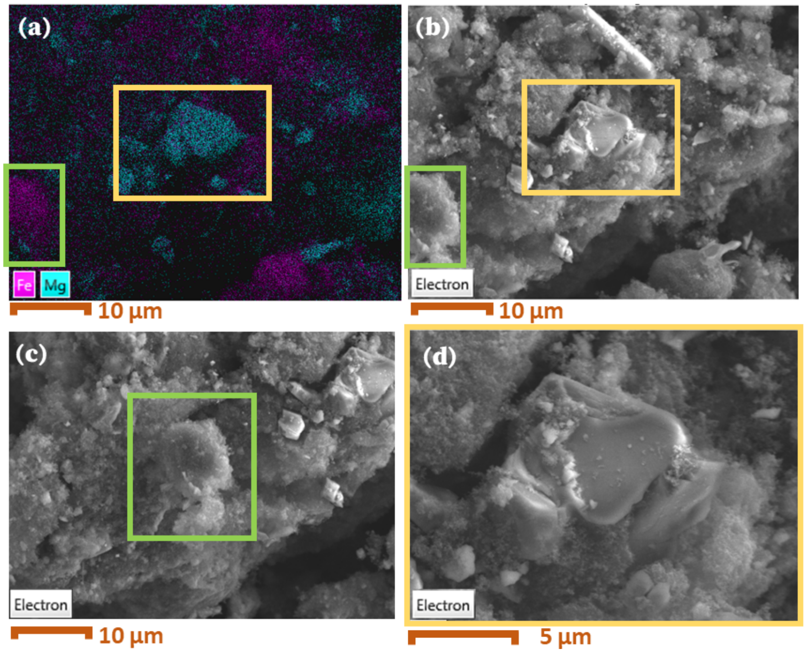

Fig. S3. a) SEM EDS map and b) corresponding SEM electron image of $MgFeB_2O_5$ cathode material **charged** to 4.2 V. c) Shifted focus SEM Electron image of the porous F features highlighted in green in a) and b). d) Zoomed-in SEM Electron image of the main cathode phase highlighted in yellow in a) and b). The Electron images show the stark contrast between the main cathode phase particles (yellow) and the porous traces of Fluorine on the conductive carbon (green), which may be deposits of reacted FeF_2.

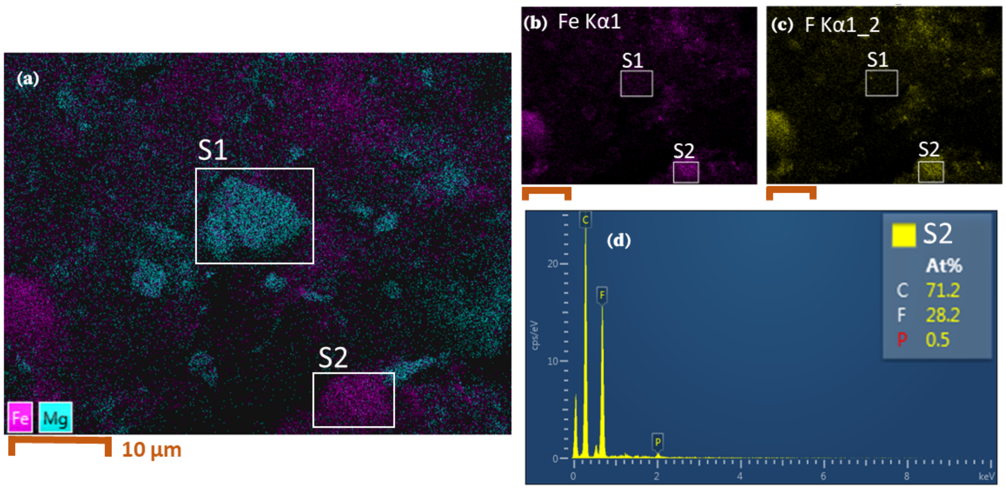

Fig. S4. SEM EDS map of $MgFeB_2O_5$ cathode material charged to 4.2 V in a coin cell. Illustrating the EDS Spectra of Region S2 - which is shown to only contain F and not Fe.

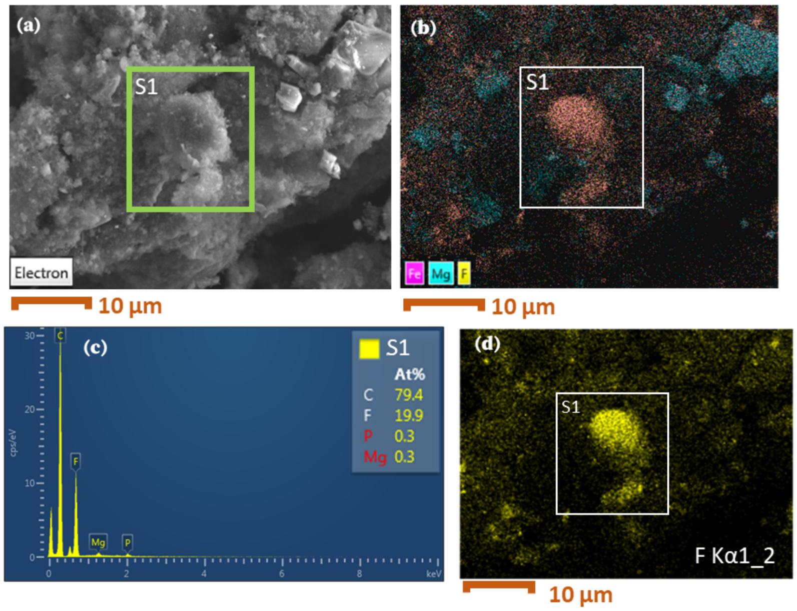

Fig. S5. a) SEM electron images and b) corresponding EDS map of porous F deposits in 4.2 V charged $MgFeB_2O_5$ cathode sample (Fig. 7 of the main paper). c) EDS Spectra of porous region demonstrating the clear presence of only F, after Fe^{2+} dissolution into the electrolyte from the deposited porous FeF_2. d) the elemental mapping image of F.

S3. *Operando* XANES Electrochemistry

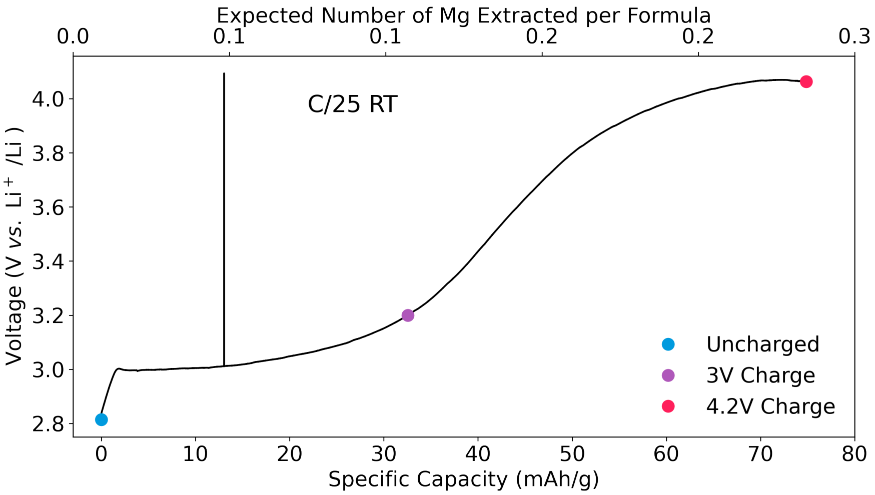

Fig. S6. Electrochemistry of *operando* cell (charged to 4.2 V at a rate of C/25 at room temperature) used for XANES measurements at the B18 beamline of Diamond Light Source. The number of moles of Mg extracted was calculated based on a theoretical capacity of 147.45 mAh g^{-1}. The coloured dots indicate the key points in the charge profile at which post-cycling analysis was performed. The Spike in the voltage is an artefact due to cable connections being nudged while the robotic arm of the beamline was moving the cell stage.

S4. Mössbauer Spectra

This section provides comprehensive fitting details of the Mössbauer spectra presented in Figures 1c and 4 of the main paper, where the data in Fig. 1c corresponds to the *uncharged* sample in Fig. 4. Fits were executed in a custom C++ script that performed convolution of the Lorentzian peaks of each individual signal. An additional Fe(II) peak was introduced as a fitting component for the 3 V charged sample to maintain continuity in the fit variables of the Fe(II) major and minor components of the cathode phase across increasing states of charge. Without this additional component, the fit variables for the 3 V charged sample were inconsistent with those for the uncharged and 4.2 V charged samples. The discontinuity in the Fe(II) cathode phase variables is illustrated in Fig. S7.

Table S4. *Ex-situ* Mössbauer spectroscopy results for $MgFeB_2O_5$ cathode samples, where δ is the Isomer Shift (mm/s) and ΔE_Q is the Quadrupole Splitting.

	Fe(II) major			Fe(II) minor		
	δ (mm/s)	ΔE_Q (mm/s)	Relative Fe(II) (%)	δ (mm/s)	ΔE_Q (mm/s)	Relative Fe(II) (%)
Uncharged	1.169(2)	2.753(13)	58(11)	1.175(5)	2.46(4)	42(8)
Charged 3 V	1.173(2)	2.755(4)	60	1.168(6)	2.468(11)	40
Charged 4.2 V	1.173(4)	2.77(3)	58(19)	1.171(9)	2.46(8)	42(19)

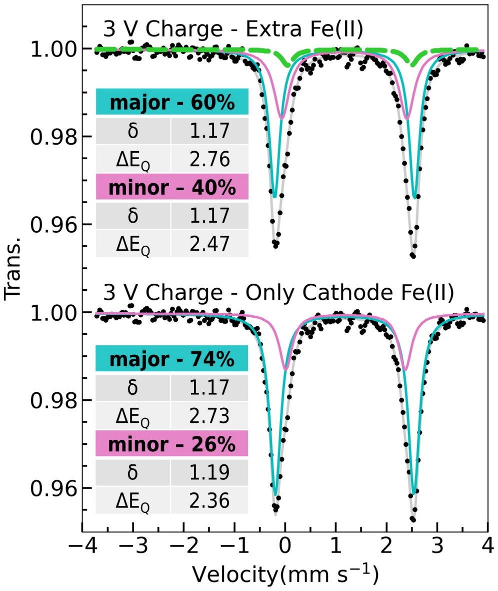

Fig. S7. *Ex-situ* ^{57}Fe Mössbauer spectra of 3V Charged $MgFeB_2O_5$ cathode sample and the final fit in grey, (top) with and (bottom) without the additional Fe(II) signal. The individual components that give rise to each fit are: the main cathode phase (i) Fe(II) major (blue) and (ii) Fe(II) minor (pink), (iii) the additional Fe(II) component (green). The inset tables show the Fe(II) cathode phase values. Here the relative Fe(II) % of each component is reported in the table title, δ is the isomer shift (mm/s) and ΔE_Q is the quadrupole splitting of each component. When comparing the values in the upper and lower inset tables with the uncharged and 4.2V charged values in table S4, the need for the additional Fe(II) peak becomes evident in maintaining continuity.

The inset table for the bottom curve shows that, in the absence of the additional Fe(II) signal,

there is a significant change in the ratio between the Fe(II) major and minor components, as well as in the quadrupole splitting for the Fe(II) minor component. Since the cathode's Fe(II) major and minor signals for the uncharged and 4.2 V charged samples agree between themselves, we do not expect such a large oscillation in the values at 3 V to be physical. The introduction of a third Fe(II) signal shows that values consistent with the uncharged and 4.2V charged fits can be obtained, top curve of Fig S7.

The fit on the 3 V data was performed by firstly fixing the two cathode Fe(II) signals to the isomer shift and quadrupole splitting refined from the uncharged data, and adding a freely evolving Fe(II) signal. This was optimised to give a percentage value of around 6.5% and a much-improved fit to the data. Due to the risk of over-fitting, the percentage of the extra Fe(II) signal was subsequently fixed to 6.5% and the isomer shifts and quadrupole splittings of all Fe(II) components were refined simultaneously. For this reason, in the tables reported here there is no error in the percentages of the Fe(II) signals for the 3 V charged sample.

Table S5. Fe metal impurity details from *ex-situ* Mössbauer Spectroscopy of $MgFeB_2O_5$ uncharged/pristine cathode sample, where δ is the Isomer Shift (mm/s).

	δ (mm/s)	Field (T)	Fe Ion (%)
Uncharged	0.00(3)	33.2(4)	11(3)

Table S6. Fit details for additional Fe(II), attributed to FeF_2, from *ex-situ* Mössbauer spectroscopy of $MgFeB_2O_5$ 3.2 V charged sample. Here δ is the Isomer Shift (mm/s) and ΔE_Q is the Quadrupole Splitting.

	δ (mm/s)	ΔE_Q (mm/s)	Fe Ion (%)
Charged 3 V	1.27(2)	2.44(4)	6.5

S5. Conversion of Mössbauer At% for SPXRD comparison

1. We start by assuming that 11 At% of Fe(0) is attributed to the Fe metal and the remaining 89 At% corresponds to the Fe(II) in our pyroborate cathode phase.

2. We then multiply the At% of the Fe components by the respective molecular weight M_i in g/mol:

$$W_i[\frac{\mathrm{g}}{\mathrm{mol}}] = \mathrm{At\%}_i \times M_i \tag{1}$$

$$W_{\mathrm{Fe}} = 11 \times 55.845 = 614.3\frac{\mathrm{g}}{\mathrm{mol}} \tag{2}$$

$$W_{\mathrm{Pyroborate}} = 89 \times 182.147 = 16211.1\frac{\mathrm{g}}{\mathrm{mol}} \tag{3}$$

Here we have assumed that there is 1 mol of Fe(II) atoms per mol of pyroborate $MgFeB_2O_5$.

3. Now that we have calculated the total weight distribution of the two components we can turn this into a weight percentage:

$$\mathrm{wt\%}_{Fe} = \frac{W_{\mathrm{Fe}}}{W_{\mathrm{Fe}} + W_{\mathrm{Pyroborate}}} \times 100 = \frac{614.3}{614.3 + 16211.1} \approx 4wt\% \tag{4}$$

4. Propagating the 3 At% error to a weight percentage error, using the following equation for $z = f(x)$

$$\Delta z = \frac{\partial x}{\partial z}\Delta x \tag{5}$$

we obtain $\Delta\mathrm{wt\%}_{Fe} = 0.959$.

Thus from the above calculations we can conclude that the (11 ± 3) At% of Fe atoms in the Fe(O) spin state identified from the Mössbauer can be converted to approximately (4 ± 1) wt% of Fe metal impurity in our phase.

S6. Calculation of expected Capacity contribution from Fe metal wt %

1. We start with Faraday's equation for the theoretical capacity, converted to units of mAh g^{-1}

$$Q = \frac{1000 \times 96485}{3600} \times \frac{z}{M} \tag{6}$$

2. From the proposed reaction pathway we expect the following half-cell equation $Fe(0) - 2e^- \longrightarrow Fe^{2+}$, thus $z = 2$

3. Given the molar mass of Cubic Fe metal is $M = 55.845$ g/mol we calculate that the theoretical capacity is

$$Q_{Fe} = \frac{1000 \times 96500}{3600} \times \frac{2}{55.845} = 959.85\frac{\mathrm{mAh}}{\mathrm{g}} \tag{7}$$

4. Now we can calculate a range of expected capacity contribution for the range of Fe metal impurity percentage present in our sample:

$$Q_{max} = 0.075 * 959.85 = 72.0\frac{\mathrm{mAh}}{\mathrm{g}} \tag{8}$$

$$Q_{min} = 0.04 * 959.85 = 38.4\frac{\mathrm{mAh}}{\mathrm{g}} \tag{9}$$

Due to the varying Fe metal composition wt% from different synthesis iterations being in the range between 4-7.5 wt% we expect a total capacity contribution in the range of $\approx 40 - 70$ mAh g^{-1} for our proposed Fe dissolution reaction.

S7. On the Irreversibility of the Fe metal reaction

Post-cycling analysis on the pyroborate was also performed beyond the first charge, to investigate both the reversibility of the proposed Fe metal reaction, as well as any further redox and intercalation behaviour of the host cathode structure when cycled vs Li.

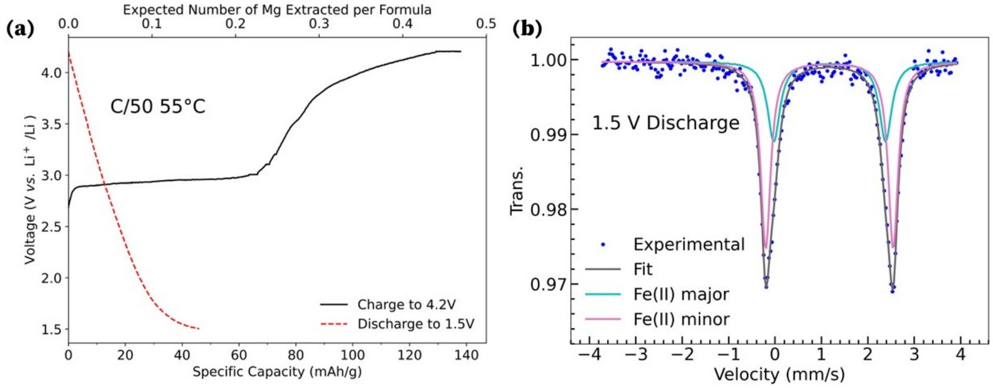

Fig. S8. a) Electrochemistry of MgFeB2O5 charged to 4.2 V with a CCCV hold and discharged to 1.5 V at a rate of C/50 in a 55 °C oven. b) *ex-situ* ^{57}Fe Mössbauer spectra of discharged $MgFeB_2O_5$ cathode sample, recorded from harvested electrode cycled in a coin cell.

Firstly, we show that the Fe metal reaction is in fact irreversible, as evidenced in figure S8 a) by the low capacity reached on the first discharge and lack of any plateaus. *ex-situ* Mössbauer spectra of the sample at the end of the first discharge were also collected as seen in figure S8 b), and clearly show the lack of Fe metal impurity in the sample and persistence of the Fe(II) high spin state. We therefore conclude that the proposed Fe metal impurity reaction is irreversible.

XANES spectra were collected at the B18 beamline of Diamond Light Source on the samples at the end of discharge. As seen in figure S9, they show no significant change in the spectral features when compared to the uncharged and 4.2 V charged spectra. This indicates that the low capacities observed after the first charge are likely due to Li plating rather than any direct reaction of the Li with the cathode phase, which would result in a change in the Fe environment.

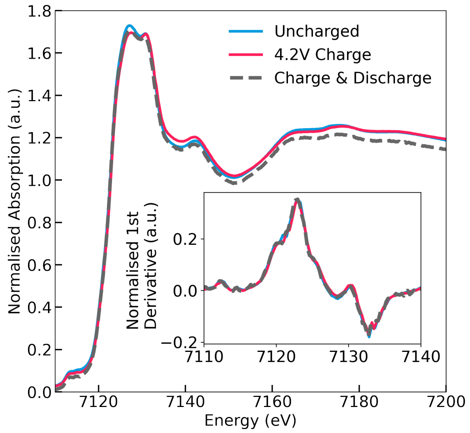

Fig. S9. Normalised Fe K-edge *operando* XANES spectra of discharged (grey) cathode compared to uncharged (blue) and end of charge at 4.2 V (red) cells. (Inset) derivative of corresponding XANES spectra.

S8. NMR

This section contains the full results for the ^{1}H and ^{19}F NMR spectra reported in the Paper in Figure 6.

Solution NMR did not show traces of H_2O present in the pristine electrolyte. In addition, Karl-Fischer titration was performed, as this is a more sensitive technique to small traces of water. Karl-Fischer titration was performed with a coulometric model, using a Metrohm Coulometer 899, and gave a reading of 13.2 ppm of H_2O, (0.5 ml of electrolyte, drift correction time 45.9 s). Confirming that the electrolyte is not the main source of water introduction to the battery system. Any water absorbed by electrolytes during storage tends to react directly with the $LiPF_6$ salt, producing $PO_xF_y^{n-}$ anions and HF, the latter being clearly detectable in our ^{1}H NMR spectrum of the pristine electrolyte, Table S7.

Table S7. Summary of Identified chemical shifts observed in the ^{1}H and ^{19}F NMR spectra in this work, their assignments and the electrolyte condition in which they were identified.

Nucleus	Shift (ppm)	Assignment	Pristine or Cycled?
^{1}H			
	4.48	EC	Both
	3.70	DMC	Both
	2.50	DMSO	Both
	10.55 (J = 410 Hz)	HF	Pristine
	0.8-1.4	DMSO impurity	Both
	3.34	Water	Cycled
	4.29	Lithium ethylene dicarbonate	Cycled
	3.18	Methanol	Cycled
	4.11	Methanol	Cycled
^{19}F			
	-74.5	PF_6^-	Both
	-83.2	$OPF_2(OCH_3)$	Pristine
	-172.2	HF	Pristine
	-82.9	Oxyfluorophosphate salts	Cycled
	-138.8	SiF_x	Cycled
	-152.8	BF_4^-	Cycled

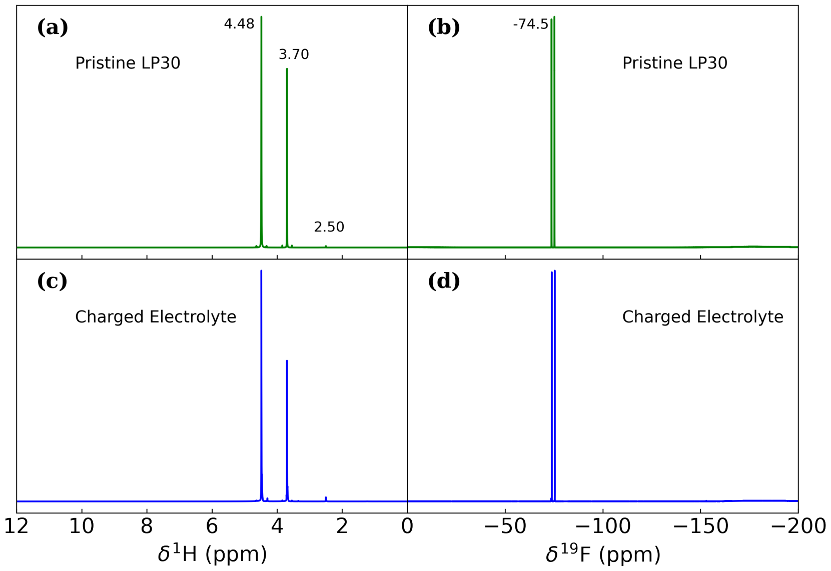

Fig. S10. Full solution NMR spectra of those shown in Figure 6 in the main text; (top left) ^{1}H spectra of a) pristine LP30 electrolyte and c) electrolyte extracted from the coin cell after 4.2 V charge (bottom left), c)^{19}F spectra of pristine LP30 electrolyte (top right) and d) (bottom right) electrolyte extracted from the coin cell after 4.2 V charge. For ^{1}H the signal of ethylene carbonate (EC; 4.48 ppm), dimethyl carbonate (DMC; 3.70 ppm) and dimethyl sulfoxide (DMSO; 2.50 ppm) are annotated on the top left spectra. For ^{19}F the signal of ethylene carbonate (PF_6^-; -74.5 ppm), is annotated on the top right spectra.

S9. ICP of Li metal Anode

The coin cells opened for Mössbauer spectroscopy were also analysed with Inductively Coupled Plasma (ICP) Spectroscopy. Cathodes from the cells were washed and shipped to Montpellier as described in the experimental section of the paper, whilst the Li metal anodes harvested in an inert atmosphere glovebox were dissolved into 12ml of millipore water. The diluted anode solutions were run and analysed on a Thermo Fisher Scientific iCAP7400 Duo ICP-OES spectrometer using Qtegra software, using ICP standards from Sigma-Aldrich and nitric acid (Trace Metal grade) from Fisher.

Due to the sticking of the Li metal to the cell spacers, there were small variations in the weights of the anodes harvested. This means that direct comparison of the ppm values between the cells should not be trusted. Nevertheless, we observe that on the anodes there are small traces of Cr, Al and Fe which we believe to be coming from standard corrosion of the stainless steel casing of the cells when cycled in an oven at 55°C.

Table S8. ICP Results of cycled Li metal anodes, at various states of Charge.

Sample	Fe ppm	Mg ppm	Al ppm	Cr ppm
Li Metal Anode 3.2V Charge	0.164	0.000	0.379	0.000
Li Metal Anode 4.2 V Charge	0.138	0.000	0.544	0.022
Li Metal Anode x2 Charge	0.197	0.002	0.674	0.023

The ICP data confirms that no Mg is being removed from the cathode phase and deposited on the anode, within machine error and sensitivity. In addition, whilst the Fe products from our proposed Fe metal dissolution reaction may be depositing on the Li metal anode, our ICP studies cannot offer conclusive proof. The small quantities of Fe ions observed on the anode could be wholly attributed to stainless steel corrosion indicated by the presence of Al and Cr.
